# Embryonic *Cul4b* is important for epiblast growth and location of primitive streak layer cells

**DOI:** 10.1371/journal.pone.0219221

**Published:** 2019-07-01

**Authors:** Chun-Yu Chen, I-Shing Yu, Chen-Hsueh Pai, Chien-Yu Lin, Shu-Rung Lin, You-Tzung Chen, Shu-Wha Lin

**Affiliations:** 1 Department of Clinical Laboratory Sciences and Medical Biotechnology, College of Medicine, National Taiwan University, Taipei, Taiwan; 2 Laboratory Animal Center, College of Medicine, National Taiwan University, Taipei, Taiwan; 3 Department of Bioscience Technology, College of Science, Chung-Yuan Christian University, Taoyuan, Taiwan; 4 Center for Nanotechnology and Center for Biomedical Technology, Chung-Yuan Christian University, Taoyuan, Taiwan; 5 Graduate Institute of Medical Genomics and Proteomics, National Taiwan University College of Medicine, National Taiwan University, Taipei, Taiwan; 6 Department of Laboratory Medicine, National Taiwan University Hospital, College of Medicine, National Taiwan University, Taipei, Taiwan; 7 Center of Genomic Medicine, National Taiwan University, Taipei, Taiwan; Laboratoire de Biologie du Développement de Villefranche-sur-Mer, FRANCE

## Abstract

*Cul4b*-null (*Cul4b*^*Δ/*^*Y*) mice undergo growth arrest and degeneration during the early embryonic stages and die at E9.5. The pathogenic causes of this lethality remain incompletely characterized. However, it has been hypothesized that the loss of *Cul4b* function in extraembryonic tissues plays a key role. In this study, we investigated possible causes of death for *Cul4b-*null embryos, particularly in regard to the role of embryonic *Cul4b*. First, we show that the loss of embryonic *Cul4b* affects the growth of the inner cell mass *in vitro* and delays epiblast development during the gastrulation period at E6.5~E7.5 *in vivo*, as highlighted by the absence of the epiblastic transcription factor *Brachyury* from E6.5~E7.5. Additionally, at E7.5, strong and laterally expanded expression of *Eomes* and *Fgf8* signaling was detected. Sectioning of these embryos showed disorganized primitive streak layer cells. Second, we observed that *Mash2*-expressing cells were present in the extraembryonic tissues of *Cul4b-*deficient embryos at E6.5 but were absent at E7.5. In addition, the loss of *Cul4b* resulted in decreased expression of cyclin proteins, which are required for the cell cycle transition from G1 to S. Taken together, these observations suggest that the embryonic expression of *Cul4b* is important for epiblast growth during E6.5~E7.5, and the loss of *Cul4b* results in either delayed growth of the epiblast or defective localization of primitive streak layer cells. As a result, the signaling activity mediated by the epiblast for subsequent ectoplacental cone development is affected, with the potential to induce growth retardation and lethality in *Cul4b*^*Δ/*^*Y* embryos.

## Introduction

During embryogenesis, pluripotent stem cells (e.g., embryonic stem cells), under precise control of DNA replication and repair, can give rise to an entire organism. At the blastocyst stage of mouse embryos, typically embryonic day 3.5 (E3.5), two distinct cell lineages contribute to early embryonic and extraembryonic tissue development [1]. These cell lineages include the inner cell mass (ICM) and the trophectoderm, respectively. The ICM develops into the embryo via an intermediate epiblast cell stage (beginning at approximately E4.0) and the extraembryonic endoderm via a primitive endoderm (PrE) stage. At E6.5~E7.5 in mouse embryos, epiblast cells undergo epithelial-mesenchymal transition (EMT) and ingress into the primitive streak during gastrulation. Epiblast cells emerging from the primitive streak between the embryonic ectoderm and overlying visceral endoderm give rise to the mesoderm [2–4]. Further development of the primitive streak gives rise to three embryonic germ layers, namely, the ectoderm, mesoderm, and endoderm. The primitive endoderm layer is eventually incorporated into the extraembryonic ectoderm to become part of the placenta.

Transcription factors play important roles in embryonic stem cell fate determination and in the regulation of embryo development. For example, at the mid-blastocyst stage (E3.75). *Oct4* regulates the development of the ICM into either the epiblast or the PrE via the regulation of FGF4 and EGF2 expression. Conversely, the loss of *Oct4* results in the failure of PrE segregation [5], which can be rescued in the presence of exogenous FGF2 or FGF4 [6]. During gastrulation, the posterior epiblast cells in the primitive streak initiate EMT. These cells express *Fgf8* as well as many other transcription factors, e.g., *Brachyury* [7]. The loss of *Fgf8* causes streak cells to migrate away from the primitive streak and thus affects gastrulation [8]. Additionally, during gastrulation, *Brachyury* is important for mesodermal morphogenetic cell movements [9]. In addition, *Eomes* expression in the epiblast is essential for the formation of the mesoderm, which starts in the posterior epiblast and extends into the primitive streak and mesoderm [10]. During the gastrulation stage, in addition to OCT4 and the FGF2/FGF4 signaling axis, there are other regulatory signaling pathways that affect both epiblast cells and the extraembryonic ectoderm to support the further proliferation and differentiation of mouse embryonic stem cells and extraembryonic tissues. For example, the *TGFβ* superfamily member, *Bmp4*, is produced by the extraembryonic ectoderm and is required for the morphogenesis of the posterior mesoderm [11–13]. In terms of extraembryonic tissue development, *Mash2* helps maintain giant cell precursors and spongiotrophoblast cells in a less-differentiated state [14], and in combination with the aforementioned transcription factors, this gene is also required for the development of extraembryonic tissues. The basic helix-loop-helix (bHLH) transcription factor, *Hand1*, is also required for the differentiation of trophoblast giant cells (TGCs), which represent a terminally differentiated endocrine cell type [15, 16]. As *Hand1* induces the differentiation of spongiotrophoblast cells into endoreduplicated giant cells, this gene is mainly expressed in the ectoplacental cone and exhibits activities that oppose those of *Mash2* [16]. Moreover, in *Hand1*-deficient mice, the abnormal formation of TGCs results in an embryonic lethal phenotype (at E7.5~8.5) [16, 17].

Mutations in a member of the X-linked *Cullin* gene family, *Cul4b*, have been associated with human X-linked intellectual disabilities [18, 19]. While CUL4B is mainly expressed in the ICM and in trophoblast cells, the expression of CUL4B has also been detected in extraembryonic tissues (e.g., the extraembryonic ectoderm, PE, ectoplacental cone, visceral endoderm, and chorionic ectoderm) during the peri-implantation blastocyst and gastrulation (E4.5~E7.5) stages of development [20, 21]. The disruption of *Cul4b* in mice by gene trapping in embryonic stem cells resulted in embryonic lethality before E9.5 [22]. Subsequently, our group [23] and others have attempted to create a conditional deletion of *Cul4b* by mating *loxP*-floxed *Cul4b* mice with several *Cre-*expression lines (e.g., *CAG-Cre* or *EIIa-Cre* mice). However, embryonic lethality was still observed. The successful deletion of *Cul4b* was achieved only when the *Sox2-Cre* mouse line was mated with *loxP*-floxed *Cul4b* mice for the selective deletion of *Cul4b* in the epiblast and not in extraembryonic tissues. *Cul4b*-null male (*Cul4b*^*Δ/*^*Y*) mice showed no obvious defects [20, 21, 23], although as adults, male *Cul4b*^*Δ/*^*Y* mice displayed behavioral defects and defects in postmeiotic sperm development [24, 25]. Consequently, previous studies inferred that the lethality of *Cul4b*^*Δ/*^*Y* was due to a complete loss of *Cul4b* in the extraembryonic tissues and that the role of embryo proper-derived *Cul4b* for embryo development was minimal [20, 21]. However, studies have shown that during early embryonic development, the proximal inner cell mass-derived epiblast maintains the proliferation of trophoblast stem cells (TSCs) [26, 27]. Accordingly, defective epiblasts that cannot promote the proliferation of TSCs will lead to a failure to maintain the extraembryonic ectoderm [28]. Therefore, in addition to the importance of extraembryonic *Cul4b* in the maintenance of embryo survival, whether embryonic tissue-derived *Cul4b* also plays a role in early embryo development remains to be investigated.

The aim of this study was to investigate the role of *Cul4b* in mouse embryo development during gastrulation. We examined embryos, extraembryonic development, and the expression levels of transcription factors and cell cycle regulators in embryonic tissues from *Cul4b*-null hemizygous (*Cul4b*^*Δ/*^*Y*) male offspring as well as their littermate controls, including heterozygous female offspring carrying the *Cul4b*-null allele derived from paternal (*Cul4b*^*+/Δ*^) or maternal (*Cul4b*^*Δ/+*^) origins. The results suggest that *Cul4b* is essential for the proper development and localization of primitive streak layer cells.

## Materials and methods

### Animals

*Cul4b* conditional KO mice carrying the *loxP*-floxed *Cul4b* allele were generated as described previously [23]. Breeding strategies are according to S1 Fig. To obtain *Cul4b* heterozygous (*Cul4b*^*+/Δ*^ and *Cul4b*^*Δ/+*^) and null (*Cul4b*^*Δ*^*/Y*) embryos using strategy A, we first generated *Cul4b*^*+/Δ*^ female mice by breeding *Cul4b*^*lox/*^*Y*;*Prm1-Cre* male mice (*Prm-Cre* deleter mice, stock no. 007252, Jackson Laboratory, Bar Harbor, ME, USA) to WT female mice. *Cul4b*^*lox/*^*Y*; *Prm1-Cre* male mice carrying the *Prm1-Cre* transgene provided Cre recombinase to excise the *loxP*-floxed allele at the spermatid stage of spermatogenesis and produced sperm bearing the *Cul4b*-null allele. The *Cul4b*^*+/Δ*^ female mice were subsequently bred with WT males to generate *Cul4b*^*Δ/+*^ female pups (S1A Fig). Strategy B was also applied to generate *Cul4b* heterozygous and null embryos (S1B Fig). The *Zp3-Cre* line [C57BL/6-Tg (Zp3-cre)93Knw/J; 003651; Jackson Laboratory, Bar Harbor, ME, USA) was purchased from the Jackson Laboratory. *Cul4b*^*lox/+*^*; Zp3-Cre* female mice carrying the *Zp3-Cre* transgene provided *Cre* recombinase to excise the *loxP*-floxed allele in the growing oocyte prior to the first meiotic division and produced oocyte bearing the *Cul4b*-null allele. The deleted allele was indicated as the deleted (null) ‘*Δ*’ allele.

All experimental procedures were approved by Institutional Animal Care and Use Committee (IACUC) of the College of Medicine, National Taiwan University (Protocol #20140289), and were performed according to its guidelines. All animals were housed in the Laboratory Animal Center of the College of Medicine of National Taiwan University (AAALAC Accredited) under a 12-h light/dark cycle with free access to food and water.

### Genotyping of mice and embryos

For genotyping of mice, genomic DNA was extracted from mouse toes by using DirectPCR Lysis Reagent (Viagen). Routine genotyping of *Cul4b* mice was performed by polymerase chain reaction (PCR) with primer in3F1 paired with primer in3R1 for the WT and floxed allele or paired with primer in5R1 for the deleted allele. The following PCR protocol was used: 94°C for 2 min; 35 cycles of 94°C for 30 s, 63°C for 1 min and 72°C for 50 s; and 72°C for 7 min. PCR primers for detection of the *Prm1-Cre* transgene were Prm1-F (5’-GCGGTCTGGCAGTAAAAACTATC-3’) paired with Prm1-R (5’-GTGAAACAGCATTGCTGTCACTT-3’). The PCR protocol was the same as for *Cul4b*. Primer sequences used in genotyping are listed in S1 Table.

For E6.5 to E7.5 embryo genotyping, pregnant females were sacrificed at the indicated embryonic day. Embryos were lysed in lysis buffer (50 mM Tris pH 8.8, 1 mM EDTA pH 8.0, 0.5% Tween 20, 200 μg/ml Proteinase K) and boiled at 95°C for 10 min. The primer set used in *Cul4b* genotyping was in3F1 (5’-CATCTTTAGC CTCTTGTGCT-3’), in3R1 (5’-AAAAGCCTACGTTTATGTGC-3’) and in5R1 (5’-AGCCTGGTCTACAAAGTTGA-3’). The primer set used in Y chromosome genotyping was Sry-F (5'-TGACTGGGATGCAGTAGTTC-3') and Sry-R (5'-TGTGCTAGAGAGAAACCCTG-3'). For genotyping wax sections containing embryos, wax was removed by rinsing with xylene for 5 min, and sections were scraped by a 27G needle. Scraped sections were lysed in lysis buffer and genotyped as mentioned above.

### Immunofluorescent staining

Embryos within deciduae were fixed in 4% paraformaldehyde, embedded in paraffin and sectioned. Dewaxed embryo sections were boiled in 0.01 M citrate buffer (pH 6.0). After blocking with Rodent Block M, sections were incubated overnight with an appropriate amount of primary antibodies against CUL4B, OCT4, CDX2, AP-2γ, EOMES and GATA4 diluted in antibody diluent at 4°C. Unbound antibodies were removed by washing in PBST three times. To visualize immunofluorescent signals, DyLight 488- and Cy3-conjugated secondary antibodies were used. Sections were cover-slipped with antifade mounting medium and examined by fluorescence microscopy.

### Morphological and histological analysis

*Cul4b* KO embryos of E6.5 and E7.5 were isolated form pregnant *Cul4b*^+/*Δ*^ heterozygous female mice after mating with WT B6 or *Cul4b*^*lox*^*/Y;Prm1-Cre* male mice. The embryos were washed in ice-cold 1X phosphate buffered saline (PBS) and fixed in 4% paraformaldehyde 4°C overnight. After washing with 1XPBS, images were taken under stereo microscope (SMZ100, Nikon) with a CCD camera (DP72, Olympus, Tokyo, Japan). For histological analysis, fixed embryos were dehydrated and embedded in paraffin, then cut into 5-μm serial sections and stained with hematoxylin and eosin. Images were captured under a microscope (DMR, Leica) equipped with a CCD camera.

### BrdU incorporation of embryos

To measure proliferation deficiency of Cul4b KO embryos, 100 mg/mg of body weight of BrdU (B5002, Sigma-Aldrich) was injected into each pregnant female mouse intraperitoneally. Thirty minutes or 2 h later, the embryos were isolated, fixed in 4% paraformaldehyde at 4°C overnight, embedded in paraffin, and then sectioned (5 μm). Incorporated BrdU was detected by anti-BrdU monoclonal antibody using the same protocol that was used for immunohistochemistry.

### Terminal deoxynucleotidyl transferase dUTP nick end labeling (TUNEL) assay

Apoptotic cells were detected using the DeadEnd Fluorometric TUNEL System (Promega, Madison, WI). Sections were rinsed with distilled H2O and 1X PBS. Then the sections were permeabilized with 200 mg/ml of Proteinase K for 15 min. The sections were incubated with equilibration buffer for 10 min at room temperature (RT). The color development reaction was performed according to the manufacturer’s recommended procedures, and the results were investigated with a microscope (DMR, Leica) equipped with a CCD camera (DP72, Olympus).

### Measurement of mitotic index

Embryonic sections were incubated overnight with primary antibodies against pHH3. To reveal the immunoreactive signals, DyLight 488- and Cy3-conjugated secondary antibodies (Jackson ImmunoResearch Laboratories, West Grove, PA, USA) were used. Sections were cover-slipped with antifade mounting medium (DakoCytomation, Carpinteria, CA, USA) and examined with a microscope (DMR, Leica) equipped with a CCD camera (DP72, Olympus). Four to six sections of a series of sections for each embryo (n = 3 for each genotype) were used. The mitotic index was measured as the ratio of pHH3-positive cells normalized with DAPI-positive cells.

### Whole-mount *in-situ* hybridization

DIG-labelling probes were synthesized with a DIG RNA labelling kit (1-175-025, Roche). We used the whole-mount *in-situ* hybridization protocol described by Gavrieli et al. [29]. Briefly, E6.5 or E7.5 embryos were fixed in fresh 4% paraformaldehyde-PBS (pH 7.4) for 2 h at room temperature and dehydrated by a series of methanol/PBST for preservation. Before proteinase K treatment, embryos were rehydrated by a methanol/PBST series, bleached with 6% H_2_O_2_ and postfixed with 4% paraformaldehyde/0.2% glutaraldehyde in PBST. Hybridization with the desired probes was done at 68°C overnight, followed by stringent washing in TBST at 68°C. Embryos were blocked in blocking reagent with 10% sheep serum and incubated with AP-conjugated anti-DIG antibody. AP activity was detected in NBT/BCIP in NTMTL staining buffer (100mM NaCl, 100mM Tris-HCl pH9.5, 50mM MgCl_2_, 1% Tween 20 and 2mM levamisol) at room temperature for 1 h to overnight depending on the hybridization probes.

### Morphological and histological analysis

*Cul4b* KO embryos of E6.5~E7.5 were washed in ice-cold 1X phosphate buffered saline (PBS) and fixed in 4% paraformaldehyde 4°C overnight. After washing with 1XPBS, images were taken under stereo microscope (SMZ100, Nikon) with a CCD camera (DP72, Olympus, Tokyo, Japan). For histological analysis, fixed embryos were dehydrated and embedded in paraffin, then cut into 5-μm serial sections and stained with hematoxylin and eosin. Images were captured under a microscope (DMR, Leica) equipped with a CCD camera. To genotype the embryos, sections were scraped by 27G needle and boild in 50mM Tris pH 8.8, 1mM EDTA pH 8.0, 0.5% Tween 20, 200μg/ml protein kinase.

### Blastocyst outgrowth

Blastocyst outgrowth study and analysis the area of blastocyst outgrowth followed the work by Yelian et al. [30]. *Cul4b*^*Δ/*^*Y* blastocysts were collected by mating WT B6 male mice with *Cul4b*^*+/Δ*^ heterozygous female mice. At E3.5, blastocysts were flushed from the uteri of female mice and individually cultured for 7 d in gelatine-coated 24-well plates in high-glucose DMEM containing 20% foetal bovine serum. Blastocysts were incubated with 5% CO_2_ and at 37°C. Growth and sizes of blastocysts were monitored and photographed daily and measured by ImageJ software.

### Immunoblotting

Embryos were dissected from pregnant females at gestational stage 8.5~9.5 and the deciduas were torn off. Total proteins were extracted with Mammalian Protein Extraction Reagent (M-PER; Thermo Scientific, Waltham, MA, USA) containing a protease inhibitor cocktail (P8340, Sigma-Aldrich). Protein concentrations were determined with a Pierce BCA protein assay kit (Thermo Scientific). Appropriate amounts of the extracts were fractionated by SDS-PAGE, electrotransferred to PVDF membranes, and detected with antibodies against CUL4B (HPA011880, Sigma-Aldrich), β-catenin (14–6765, ebioscience, San Diego, CA, USA), phospho-β-catenin (S33/S37/T41) (9561, Cell Signaling Technology, Danvers, MA, USA), cyclin D1 (ab10540, Abcam, Cambridge, MA, USA), cyclin D2 (ab3087, Abcam), cyclin D3 (ab28283, Abcam), cyclin E (630701, Biolegend, San Diego, CA, USA), and β-actin (A5060, Sigma-Aldrich). Signals from the reaction with anti-mouse IgG and anti-rabbit IgG horseradish peroxidase (HRP)-conjugated secondary antibodies (AP124P and AP132P, Millipore) were developed with the Immobilon Western Chemiluminescent HRP substrate (WBKLSO500, Millipore). The signals were semiquantified using Multi Gauge V3.0 software (Fujfilm) and normalized against that of β-actin.

### Statistical analysis

All data were analysed using GraphPad Prism 5 software. A two-tailed unpaired Student’s *t*-test or one-way analysis of variance (ANOVA) followed by post hoc Bonferroni’s multiple comparison tests were performed to compare data, with a *P*-value < 0.05 being considered statistically significant.

## Results

### The role of CUL4B expression in embryonic tissues in the growth of mouse embryos at E6.5~E7.5

**Fig 1:**
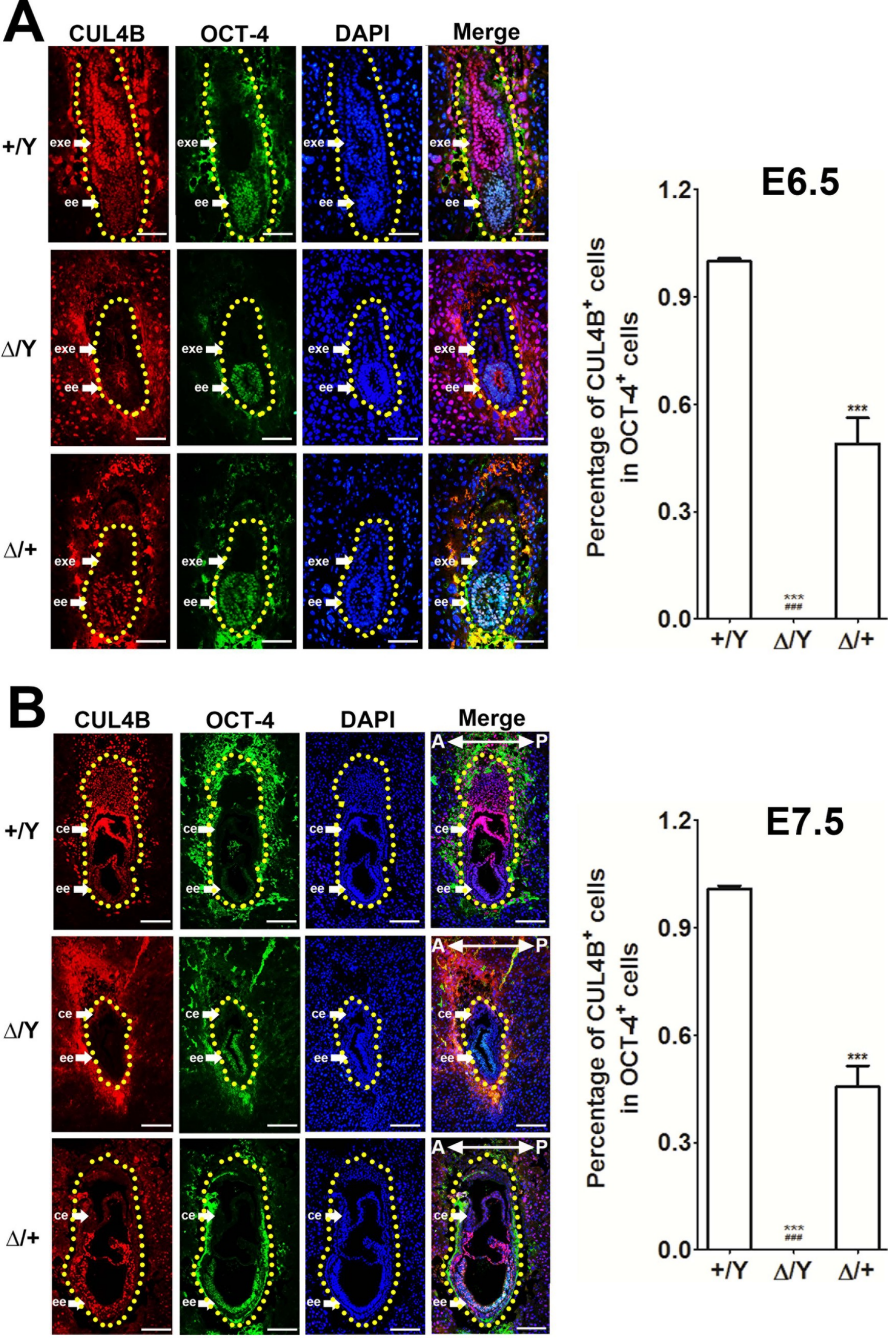
Expression pattern of CUL4B embryos at E6.5 and E7.5. (A) Immunostaining was performed for CUL4B (red), the embryonic ectoderm marker OCT3/4 (green), and the nuclear marker DAPI (blue) in E6.5 Cul4b WT (*Cul4b*^*+/*^*Y*, +/Y), knockout (*Cul4b*^*Δ/*^*Y*, Δ/Y), and heterozygous (*Cul4b*^*Δ/+*^, Δ/+) embryos. Staining of CUL4B is observed in the extraembryonic ectoderm (exe) and the embryonic ectoderm (ee) of WT embryos and in the ee of *Cul4b*^*Δ/+*^embryos with random inactivation of X chromosomes. The CUL4B signal was not detected in either the ee or exe of *Cul4b*^*Δ/*^*Y* embryos. Due to paternal X-inactivation in the extraembryonic tissue of *Cul4b*^*Δ/+*^embryos, the CUL4B signal was barely detectable. Scale bar: 50 μm. More than three embryos per genotype were analyzed for each experiment. (B) Immunostaining of E7.5 embryos was performed. Colors and symbols are the same as described in (A). *Cul4b*^*Δ/+*^ embryos exhibited random CUL4B signals in the ee and only slight expression of CUL4B in the chorionic ectoderm (ce) due to paternal X-inactivation in extraembryonic tissue. No CUL4B protein signal was detected in the ee or ce of *Cul4b*^*Δ/Y*^ embryos, and this effect was accompanied by a reduction in embryo size. A trace amount of cells showing *CUL4B staining in a fraction of CE cells* might represent a few extraembryonic mesoderm cells that escape the random X-chromosome inactivation of *Cul4b* expression in *Cul4b*^*Δ/+*^ embryos. The upper (E6.5) and lower (E7.5) right panels show percentage of CUL4B-positive cells within the OCT4-positive cells in ee of *Cul4b*^*+/*^*Y*, *Cul4b*^*Δ/*^*Y* and *Cul4b*^*Δ/+*^ embryos, respectively. The red (CUL4B), green (OCT4), and blue (DAPI) fluorescence signals within the dotted areas were visually counted individually. All embryos were generated from *Cul4b*^*+/Δ*^ females mated with *Cul4b*^*+/Y*^ WT males or *Cul4b*^*lox/*^*Y*;*Prm1-Cre* males. More than three embryos per genotype were analyzed for each experiment. Scale bar: 100 μm. Data are expressed as means + standard errors of the mean. ***: p<0.001 was compared with *+/Y* group. ^###^: p <0.001 was compared with *Δ/+* group.

Studies have previously demonstrated that *Cul4b* heterozygous female mice (*Cul4b*^*Δ/+*^) generated from *Cul4b*^*flox/+*^ females carrying the *Cul4b*-null X chromosome from their mother crossed with *EIIa-Cre* or *CAG-Cre* male mice can survive up to E13.5 [20, 21]. To achieve different tissue specificities to clarify the timing of the lethality of *Cul4b*-null mice and to determine whether Cre activity could influence the survival of *Cul4b*^*Δ/+*^ female mice, we set up different mating strategies to produce *Cul4b*^*Δ/+*^ female mice either using *Cre* activity (*Zp3-Cre* oocyte-specific knockout mice bred with wild type male, S1B Fig) or not (*Cul4b*^*+/Δ*^ female mice bred with wild type male, S1A Fig). Our breeding data suggested that all *Cul4b*^*Δ/*^*Y* embryos died by E9.5. Moreover, the *Cul4b*^*Δ/+*^ female mice from the two different breeding strategies could survive up to E13.5 or beyond (Table 1 and Table 2), indicating that the *Cre* activity and tissue specificity had no influence. We subsequently investigated early gastrulation embryos for the possible role of embryo proper-derived CUL4B in support of the survival of *Cul4b*^*Δ/+*^ embryos beyond E9.5. We observed that the *Cul4b*^*Δ/*^*Y* embryos exhibited growth retardation at E6.5 and E7.5, while the *Cul4b*^*Δ/+*^ embryos were similar to the wild type (WT) *Cul4b*^*+/*^*Y* embryos. We used two immunostaining methods to detect CUL4B and showed that while there were prominent random CUL4B signals in the embryonic tissues of the female *Cul4b*^*Δ/+*^ embryos at E6.5~E7.5, the CUL4B signals were barely detected in the extraembryonic tissues (including the extraembryonic ectoderm and visceral endoderm) and in TGCs of these *Cul4b*^*Δ/+*^ embryos, which was similar to the staining pattern of the complete absence of CUL4B signals in their *Cul4b*-null male littermates *(Cul4b*^*Δ*^*/Y*) (Fig 1 and S2 Fig). Thus, we confirmed that the embryonic tissues of the *Cul4b*^*Δ/+*^ mice consistently express CUL4B but extraembryonic tissues were barely detectable.

**Table 1:** Genotype analysis of breeding of *Cul4b*^*lox/+*^*; Zp3-Cre* females mated with WT males*.

time	# offspring	Genotypes				
		*+/+*	*Δ/+*	*+/Y*	*Δ/Y*	absorbed
P21	182	84 (46%)	4 (2%)	94 (52%)	0 (0%)	-
E18.5	128	25 (20%)	6 (5%)	43 (34%)	0 (0%)	54 (42%)
E17.5	113	31 (27%)	10 (9%)	33 (29%)	0 (0%)	39 (35%)
E16.5	113	33 (29%)	13 (12%)	31 (27%)	0 (0%)	36 (32%)
E15.5	132	33 (25%)	16 (12%)	37 (28%)	0 (0%)	46 (35%)
E14.5	130	28 (22%)	20 (15%)	27 (21%)	0 (0%)	55 (42%)
E13.5	137	33 (24%)	30 (22%)	26 (19%)	0 (0%)	48 (35%)
E12.5	113	32 (28%)	22 (19%)	22 (19%)	0 (0%)	37 (33%)
E11.5	99	21 (21%)	25 (25%)	20 (20%)	0 (0%)	33 (33%)
E10.5	103	25 (24%)	28 (27%)	25 (24%)	0 (0%)	25 (24%)
E9.5	62	12 (19%)	15 (24%)	18 (29%)	2 (3%)	15 (24%)
E8.5	28	6 (21%)	11 (39%)	5 (18%)	4 (14%)	2 (7%)
E7.5	32	6 (19%)	7 (22%)	9 (28%)	3 (9%)	7 (22%)
E6.5	23	3 (13%)	4 (17%)	10 (43%)	4 (17%)	2 (9%)
Expected ratios		1 (25%)	1 (25%)	1 (25%)	1 (25%)	-

*: This breeding strategy was used to dissect the time of the lethality of the *Cul4b*-null embryos, including *Cul4b*^*Δ/+*^ (*Δ/+*) and *Cul4b*^*Δ/*^*Y* (Δ/Y) mice.

**Table 2:** Offspring of *Cul4b*^*Δ/+*^ heterozygous female mice generated from *Cul4b*^*+*^*/Y* males mated with *Cul4b*^*+/Δ*^ females*.

	Total P1 offspring/ total litters (mean litter size)	Genotype and no. of offspring of *Cul4b*^*+*^*/Y* males mated with *Cul4b*^*+/Δ*^ females			
		*+/Y*	*Δ/Y*	*+/+*	*Δ/+*
	136/33 (4.1)	68	0	56	12
No. expected		34	34	34	34

*: This breeding strategy was used to dissect the birth rate of the *Cul4b*^*Δ/+*^ mice.

Next, we examined the maintenance of pluripotency in the embryonic tissues of WT and *Cul4b*-mutant embryos by staining for the expression of OCT4. At embryo stages E6.5~E7.5, WT (*Cul4b*^*+/*^*Y*) and mutant *Cul4b* embryos (*Cul4b*^*Δ/+*^ and *Cul4b*^*Δ/*^*Y*) exhibited similar OCT4 expression patterns (Fig 1A and 1B). These results suggest that the loss of *Cul4b* does not affect the pluripotency of embryonic tissues at E6.5~E7.5. Moreover, we performed a bromodeoxyuridine (BrdU) incorporation assay (S4 Fig), a TUNEL assay (S5 Fig) and phosphohistone-H3 (pHH3) immunostaining for the mitotic index (S6 Fig) and Ki-67 immunostaining for the proliferative index (S7 Fig). We did not observe a phenotype of decreased cell proliferation ability or increased cell apoptosis that could explain the size reduction observed in *Cul4b* knockout (KO) embryos.

### Mutant *Cul4b*^*Δ/*^*Y* embryos failed to maintain extraembryonic tissues at E7.5

**Fig 2:**
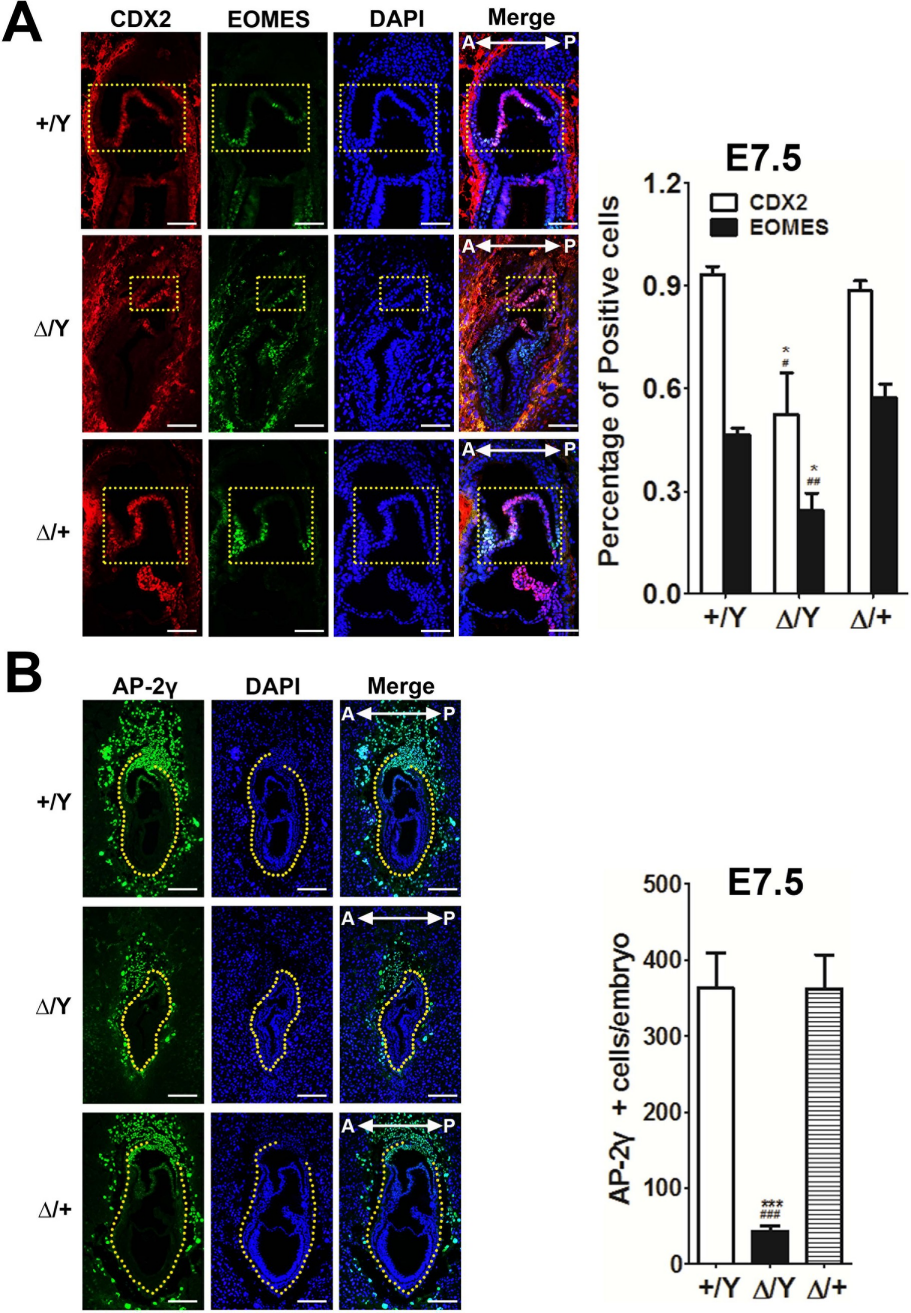
Analysis of extraembryonic tissues of E7.5 embryos. (A) Immunostaining for the pluripotent markers CDX2 (red) and EOMES (green) was performed on sections of the chorionic ectoderm (ce) from *Cul4b*^*+/*^*Y*, *Cul4b*^*Δ/*^*Y*, and *Cul4b*^*Δ/+*^ embryos (E7.5). Yellow squares indicate the cells in the chorionic ectoderm that show positive staining. Nuclei are stained with DAPI (blue). Scale bar: 100 μm. Shown on the right panel is the visually counted CDX2 and EOMES positive cells normalized with respective DAPI (blue) positive cells in the ce of *Cul4b*^*+/*^*Y*, *Cul4b*^*Δ/*^*Y* and *Cul4b*^*Δ/+*^ embryos, respectively. (B) Immunostaining of the extraembryonic lineage marker, AP-2γ (green), in *Cul4b*^*+/*^*Y*, *Cul4b*^*Δ/*^*Y*, and *Cul4b*^*Δ/+*^ embryos (E7.5). Nuclei are stained with DAPI (blue). Cells expressing AP-2γ outside the yellow region are considered to be within a region comprising TGCs. The *Cul4b* genotype for embryonic (bottom) and extraembryonic (top) tissues are marked separately by (+) for WT and (Δ) for KO. The *Cul4b*^*Δ/*^*Y* embryos contain fewer TGCs, while the *Cul4b*^*Δ/+*^ embryos contain similar numbers of TGCs as the *Cul4b*^*+/*^*Y* embryos at E7.5. All embryos were generated from *Cul4b*^*Δ/+*^ females mated with *Cul4b*^*+/*^*Y* WT males or *Cul4b*^*lox/*^*Y*;*Prm1-Cre* males. More than three embryos per genotype were analyzed for each experiment. Scale bar: 200 μm. Shown on the right panel is the visually counted AP-2γ positive cells normalized with the number of respective DAPI (blue) positive cells outside the yellow region of *Cul4b*^*+/*^*Y*, *Cul4b*^*Δ/*^*Y* and *Cul4b*^*Δ/+*^ embryos, respectively. Data are expressed as means + standard errors of the mean. *: p <0.05 and ***: p <0.001 were compared with *+/Y* group. ^#^: p <0.05, ^##^: p <0.01 and ^###^: p <0.01 were compared with *Δ/+* group.

We next examined the extraembryonic tissues. The maintenance of progenitors in the extraembryonic ectoderm requires the transcription factors *Cdx-2* and *Eomes* [10]. These progenitors develop into the chorionic ectoderm and simultaneously differentiate into the ectoplacental cone to become a source of TGCs. Therefore, we costained the extraembryonic tissues of the mutant (*Cul4b*^*Δ/*^*Y* and *Cul4b*^*Δ/+*^) embryos for CDX2 and EOMES. Both pluripotency markers were detected in WT and mutant embryos at E7.5, although compared to the WT (*Cul4b*^*+/*^*Y*) embryos, the *Cul4b*^*Δ/*^*Y* embryos contained fewer cells expressing these two markers, especially in the chorionic ectoderm (Fig 2A). In contrast, the staining patterns for CDX2 and EOMES in the chorionic ectoderm of the *Cul4b*^*Δ/+*^ embryos were unaffected (Fig 2A). Next, we examined TGCs. During gastrulation, the AP-2γ transcription factor is upregulated in TGCs and in precursor cells of the ectoplacental cone. This upregulation is important for maintaining TSCs and trophectoderm derivatives [10]. AP-2γ staining revealed that while both *Cul4b*^*Δ/+*^ and *Cul4b*^*+/*^*Y* embryos exhibited similar staining patterns, the *Cul4b*^*Δ/*^*Y* embryos contained fewer ectoplacental cone cells and secondary TGCs than did the *Cul4b*^*Δ/+*^ and WT embryos (Fig 2B). To discriminate whether the loss of the CDX2 signal in mutants results from a loss of extraembryonic fate or from a loss of its gene expression in the extraembryonic tissues of mutants, we costained the embryos for CDX2 and AP-2γ. We found increased numbers of AP-2γ single-positive cells that were CDX2 negative (S3 Fig). Therefore, we conclude that the decrease in TSCs in the extraembryonic ectoderm and chorionic ectoderm is attributed to the loss of CDX2 expression in TSCs.

### Loss of *Cul4b* in the epiblast delays the formation of the primitive streak and disrupts the localization of primitive streak layer cells

**Fig 3:**
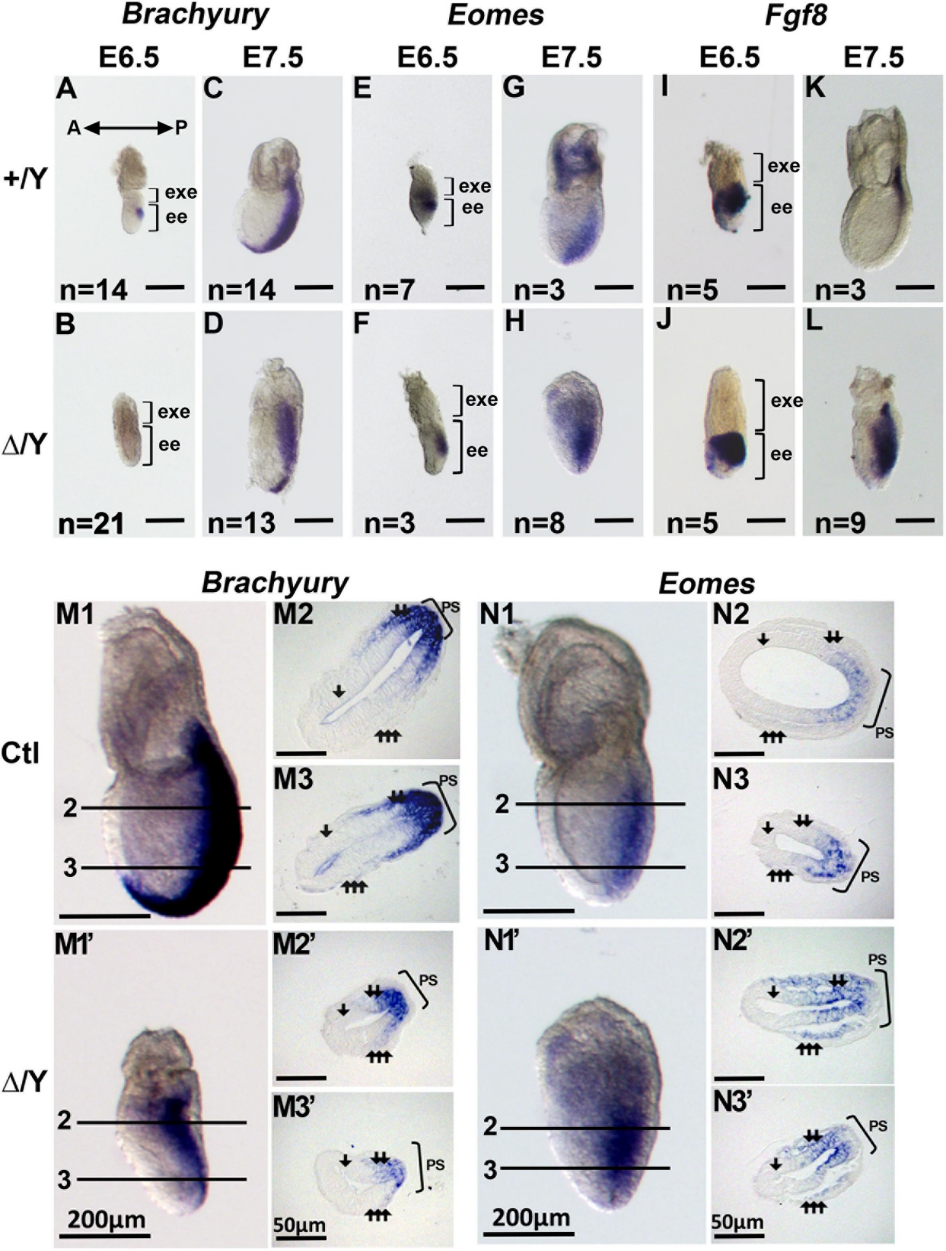
Expression of mesoderm and primitive streak markers in *Cul4b*-null embryos. Transcripts of Brachyury (A-D), Eomes (E-H), and Fgf8 (I-L) were detected in *Cul4b*^*+/*^*Y* and *Cul4b*^*Δ/*^*Y* embryos by whole-mount RNA *in situ* hybridization experiments. (A, B) At E6.5, transcripts of the primitive streak marker, Brachyury, were detected in the nascent primitive streak of *Cul4b*^*+/*^*Y* embryos but not in the primitive streak of *Cul4b*^*Δ/*^*Y* embryos. (C, D) A slight decrease in Brachyury was detected at E7.5 in *Cul4b*^*Δ/*^*Y* embryos. (G, H) Expanded expression of Eomes was detected in the primitive streak of *Cul4b*^*Δ/*^*Y* embryos compared to that of Cul4b+/Y embryos. (I, J) Transcript levels of Fgf8 in *Cul4b*^*+/*^*Y* embryos at E6.5 were similar to those in *Cul4b*^*+/*^*Y* embryos. (K, L) Transcript levels of Fgf8 in *Cul4b*^*+/*^*Y* and *Cul4b*^*Δ/*^*Y* embryos at E7.5. Scale bar: 200 μm. (M, N) Sections of whole-mount RNA *in situ* hybridizations performed for E7.5 embryos. Expanded expression of Eomes was detected in *Cul4b*^*Δ/*^*Y* embryos (N1’-N3’) compared with that in their control littermates (*Cul4b*^*+/Δ*^) (N1-N3). Sections represent distances of 175 μm and 77 μm (M2 and M3), 126 μm and 49 μm (M2’ and M3’), 182 μm and 84 μm (N2 and N3), and 140 μm and 84 μm (N2’ and N3’), respectively, from the bottom of the embryos examined. Bracket: Ctl, Control; PS, primitive streak; arrow: ectoderm, double arrow: mesoderm, triple arrow: visceral endoderm. All embryos were generated from *Cul4b*^*+/Δ*^ females mated with *Cul4b*^*+/*^*Y* WT males or *Cul4b*^*lox/*^*Y;Prm1-Cre* males.

Induction of the primitive streak is coordinated by crosstalk between the epiblast and extraembryonic ectoderm at E6.5 through the expression of developmental genes [7, 31]. *Eomes* expression in epiblasts is essential for mesoderm formation, which starts in the posterior epiblast and extends into the primitive streak and mesoderm [10]. *Fgf8* is expressed in epiblast cells undergoing EMT in the primitive streak [7]. To resolve the lineage-specific cell-type changes that occur during embryonic development, we examined the expression of specific markers of the epiblast and primitive streak, including *Brachyury*, *Eomes*, and *Fgf8*, in whole-mount *in situ* hybridization experiments. We showed that *Brachyury* was not detected during gastrulation onset in *Cul4b*^*Δ/*^*Y* embryos at E6.5 (Fig 3B), but began to appear in *Cul4b*^*Δ/*^*Y* embryos by E7.5 (Fig 3D), although the *Brachyury* staining pattern was abnormal and reduced in most embryos (S8 Fig). The abnormal *Brachyury* pattern included extended signals to the superior or anterior part of embryos (S8 Fig).

Whole-mount *in situ* hybridization for *Eomes* and *Fgf8* expression in E6.5-E7.5 *Cul4b*^*+/*^*Y* embryos revealed expression patterns consistent with those described in the literature [32–35]. *Eomes* was expressed in the primitive streak, mesoderm, and chorionic ectoderm (Fig 3E and 3G). *Fgf8* staining was localized to the primitive streak, where it first became apparent (Fig 3I) and gradually formed a gradient, showing higher staining at the posterior end and lower staining at the anterior end of the streak, with the most strongly labeled cells situated at the base of the allantois (Fig 3K). In contrast, in *Cul4b*^*Δ/*^*Y* embryos, although both *Eomes* and *Fgf8* signals were expressed from E6.5 (Fig 3F and 3J, respectively) to E7.5 (Fig 3H and 3L, respectively), these signals were stronger than the respective signals in *Cul4*^*+/*^*Y* embryos at E7.5 and revealed a lateral expansion pattern with accumulated expression at the posterior end of the primitive streak.

Sections of representative embryos revealed that the three layers (ectoderm/mesoderm/visceral endoderm) were not compact and fewer mesoderm cells were present in *Cul4b*^*Δ/*^*Y* embryos than in the control embryos (Fig 3M and 3N vs 3M’and 3N’). Moreover, mesoderm cells positive for *Eomes* transcripts were also found to be embedded in the anterior areas of the embryos (Fig 3N2’ and 3N3’) rather than having a more condensed distribution as observed in the control embryos.

**Fig 4:**
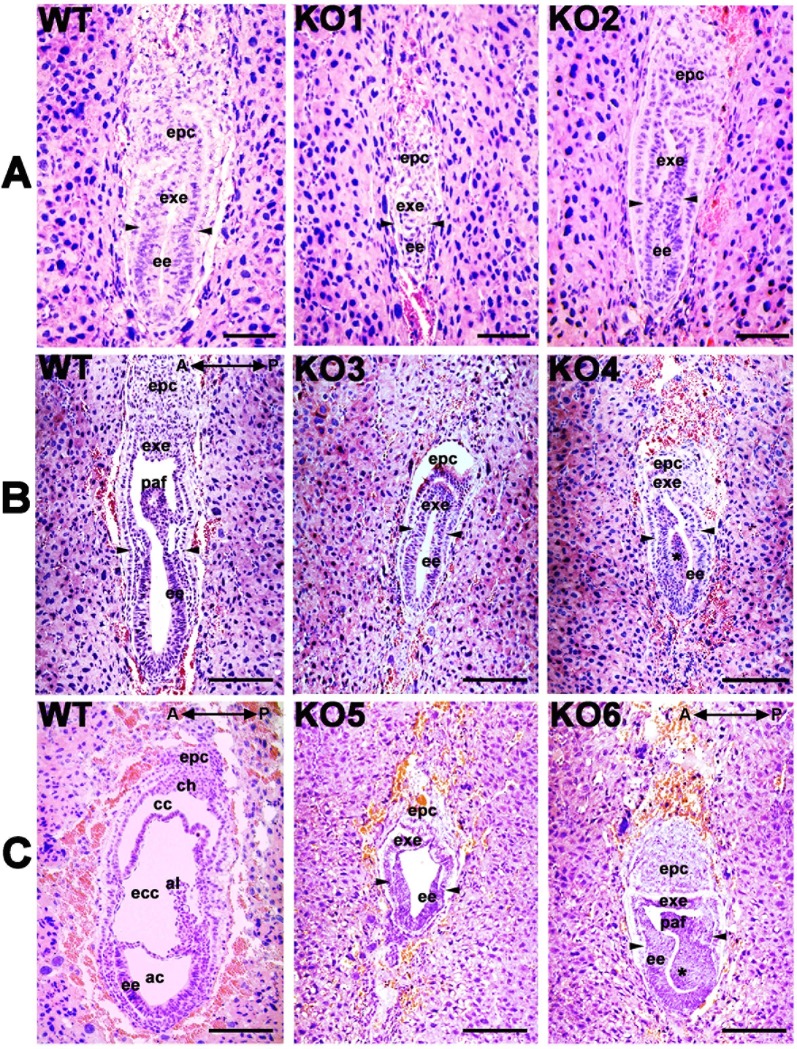
Histology of the embryos at E6.5, E7.0 and E7.5. (A) Representative embryos at E6.5, and *Cul4b*^*Δ/*^*Y* (KO1, KO2) embryos displayed a slightly smaller size than WT embryos. Arrowheads indicate the boundary of ee and exe. ee, embryonic ectoderm; exe, extraembryonic ectoderm; epc, ectoplacental cone. Scale bar: 50 μm. (B) Representative embryos at E7.0, WT embryos underwent the formation of the posterior amniotic fold (paf). *Cul4b*^*Δ/*^*Y* (KO3, KO4) embryos displayed a phenotype of delayed morphology or had abnormal paf (*). Arrowheads indicate the boundary of ee and exe. Scale bar: 100 μm. (C) Representative embryos at E7.5. WT embryos underwent the formation of three cavities, including the amniotic cavity (ac), the exocoelomic cavity (eec), and the chorionic cavity (cc). *Cul4b*^*Δ/*^*Y* (KO5, KO6) embryos displayed a single proamniotic cavity or formation of a posterior amniotic fold (paf) with accumulation of epiblasts (*). All embryos were generated from *Cul4b*^*+/Δ*^ females mated with *Cul4b*^*+/*^*Y* WT males or *Cul4b*^*lox/*^*Y;Prm1-Cre* males. Arrowheads indicate the boundary of ch, chorionic ectoderm; al, allantois. Scale bar: 100 μm.

Sectioning and H&E staining also revealed that *Cul4b*^*Δ/*^*Y* embryos had delayed appearance, and defective formation of the three cavities (Fig 4). Moreover, there seemed to be an accumulation of epiblasts at the posterior end of the *Cul4b*^*Δ/*^*Y* embryos, which is similar to the appearance observed in stained whole-mount sections. In combination, these data indicate that the loss of *Cul4b* manifests as a developmental delay, which is accompanied by changes in the localization of the primitive streak and mesodermal layer cells.

### The role of *Cul4b* in the growth of the ICM and epiblasts of the embryo proper

**Fig 5:**
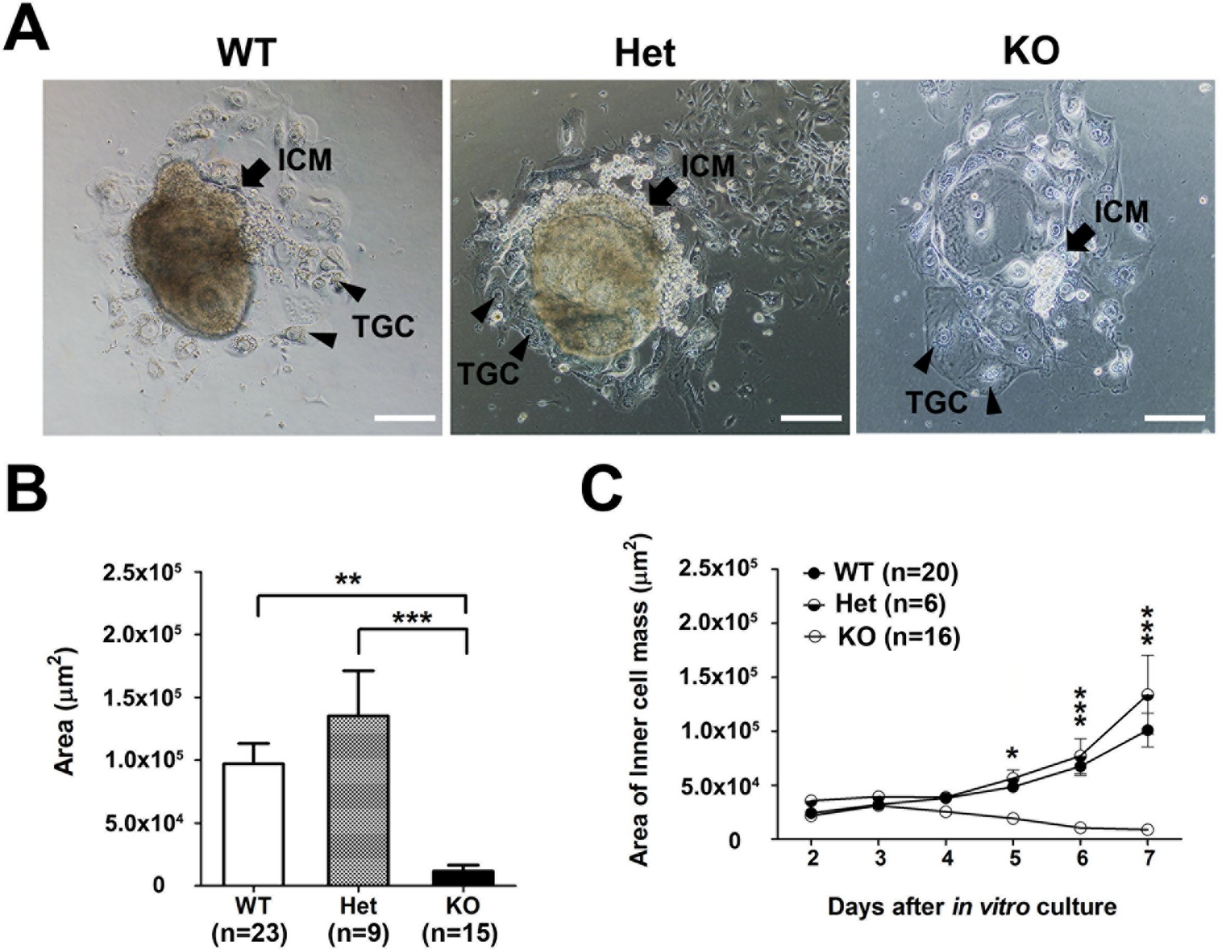
Loss of *Cul4b* in the ICM formed from blastocysts cultured *in vitro* causes proliferation defects. (A) Images of cultured *Cul4b* WT, Het, and KO blastocysts at day 7 are shown. (B) Growth areas in the ICM of *Cul4b* KO blastocysts were severely decreased compared to the WT and Het blastocysts. (C) A comparison of ICM growth rates among the *Cul4b* WT, Het, and KO blastocysts suggests that deficiencies in *Cul4b* lead to proliferation defects. All embryos were generated from *Cul4b*^*+/Δ*^ females mated with *Cul4b*^*+/*^*Y* WT males or *Cul4b*^*lox/*^*Y;Prm1-Cre* males. More than three independent experiments were performed. WT (*Cul4b*^*+/*^*Y* and *Cul4b*^*+/+*^); Het, heterozygous (*Cul4b*^*Δ/+*^ and *Cul4b*^*+/Δ*^); KO (*Cul4b*^*Δ/*^*Y* and *Cul4b*^*Δ/Δ*^); TGCs, trophoblast giant cells (arrowheads). Scale bar: 100 μm.

During early embryonic development, the epiblast maintains the proliferation of the precursor TSCs of extraembryonic tissues [26, 27]. Therefore, we performed a blastocyst outgrowth study to measure the proliferation capacity of the ICM, which gives rise to the epiblast [30]. Smaller areas of ICM proliferation were observed in *Cul4b*-null age-matched embryos (KO, *Cul4b*^*Δ*^*/Y* and *Cul4b*^*Δ/Δ*^) than in their WT (*Cul4b*^*+/*^*Y* and *Cul4b*^*+/+*^) and heterozygous (*Cul4b*^*Δ/+*^and *Cul4b*^*+/Δ*^) female age-matched embryos (Fig 5A and 5B). The impaired growth of the ICM in *Cul4b*-null embryos was first observed after five days of *in vitro* culture (equivalent to E7.5 *in vivo*), and the ICMs were not viable (Fig 5C). Additionally, the TGCs were comparable among all of the embryo genotypes examined, suggesting that the differentiation of TGCs *in vitro* is not affected by the loss of *Cul4b*. Taken together, these results indicate that the partial loss of *Cul4b* does not affect the growth of the ICM, while the complete loss of *Cul4b* does affect ICM growth. Moreover, these results are consistent with the growth retardation observed for *Cul4b*^*Δ*^*/Y* embryos but not for their heterozygous littermates (*Cul4b*^*Δ/+*^*and Cul4b*^*+/Δ*^) at E7.5. To examine whether the differentiation of TGCs *in vitro* is affected by the loss of *Cul4b*, we performed *Cul4b* knockdown in TSCs. We found that the *Cul4b****-***knockdown TSCs displayed normal proliferative ability (S10 Fig). These results suggest that the growth failure of TSCs in the extraembryonic ectoderm of *Cul4b*^*Δ*^*/Y* mice is not caused by intracellular factors. We suggested that the reduced size of the extraembryonic ectoderm in *Cul4b*^*Δ*^*/Y* embryos may have been due to a failure to maintain the crosstalk between embryonic–extraembryonic tissues.

### Loss of *Cul4b* causes a failure to maintain giant cell progenitors

**Fig 6:**
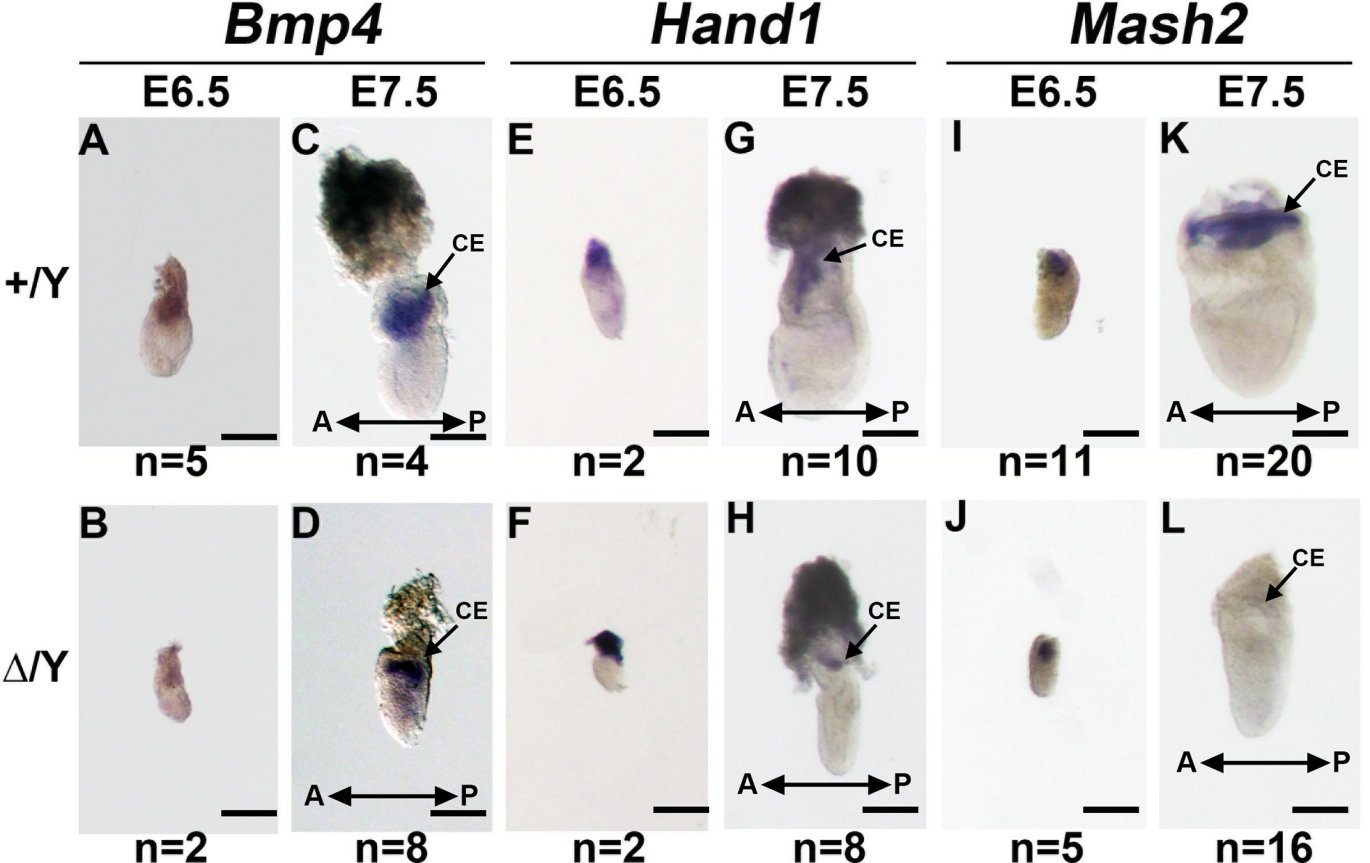
Expression of extraembryonic ectoderm and ectoplacental cone markers in *Cul4b*-null embryos. Transcripts of *Bmp4* (A-D), *Hand1* (E-H), and *Mash2* (I-L) were detected in *Cul4b*^*+/*^*Y* and *Cul4b*^*Δ/*^*Y* embryos in whole-mount RNA *in situ* hybridization experiments. At E6.5 and E7.5, the transcript levels of the extraembryonic ectoderm marker *Bmp4* and the ectoplacental cone marker *Hand1* cannot be discriminated between *Cul4b*^*+/*^*Y* and *Cul4b*^*Δ/*^*Y* embryos (A-H). The expression levels of the extraembryonic ectoderm and ectoplacental cone marker *Mash2* were not distinct between *Cul4b*^*+/*^*Y* and *Cul4b*^*Δ/*^*Y* embryos at E6.5 (I, J). A severe reduction in the chorionic ectoderm in *Cul4b*^*Δ/*^*Y* embryos was observed compared to that in *Cul4b*^*+/*^*Y* embryos at E7.5 (K, L). All embryos were generated from *Cul4b*^*+/Δ*^ females mated with *Cul4b*^*+/*^*Y* WT males or *Cul4b*^*lox/*^*Y;Prm1-Cre* males. Scale bar: 200 μm.

We further investigated whether the loss of *Cul4b* in extraembryonic tissues affects the expression of transcription factors and whether this effect correlates with the observed reduction in the number of TGCs in the extraembryonic ectoderm in *Cul4b*^*Δ/*^*Y* mutant embryos. *In situ* hybridization experiments detected the prominent expression of *Bmp4* transcripts at E7.5 in both *Cul4b*^*+/*^*Y* and *Cul4*^*Δ/*^*Y* embryos (Fig 6A–6D). These results suggest that the loss of *Cul4b* does not affect the ability of the extraembryonic ectoderm to regulate the development of the posterior mesoderm during gastrulation. In addition, the levels of *Mash2* and *Hand1* transcripts did not differ in the extraembryonic ectoderms of *Cul4*^*+/*^*Y* and *Cul4b*^*Δ/*^*Y* embryos at E6.5 (Fig 6E and 6F, and 6I and 6J, respectively). However, although *Hand1* transcription did not differ between *Cul4*^*+/*^*Y* and *Cul4b*^*Δ/*^*Y* embryos at E7.5 (Fig 6G and 6H), *Mash2* transcription in the chorionic ectoderm was markedly reduced in *Cul4b*^*Δ/*^*Y* embryos compared with that in *Cul4*^*+/*^*Y* and *Cul4b*^*Δ/+*^ embryos (Fig 6K and 6L). Taken together, these results further support the finding that giant cell progenitors are not maintained in the chorionic ectoderm of *Cul4b*^*Δ/*^*Y* embryos at E7.5, and which corresponds with the observed reduction in the number of TGCs detected with AP-2γ staining (Fig 2B and S3 Fig).

### The loss of *Cul4b* does not affect the formation of the visceral endoderm

**Fig 7:**
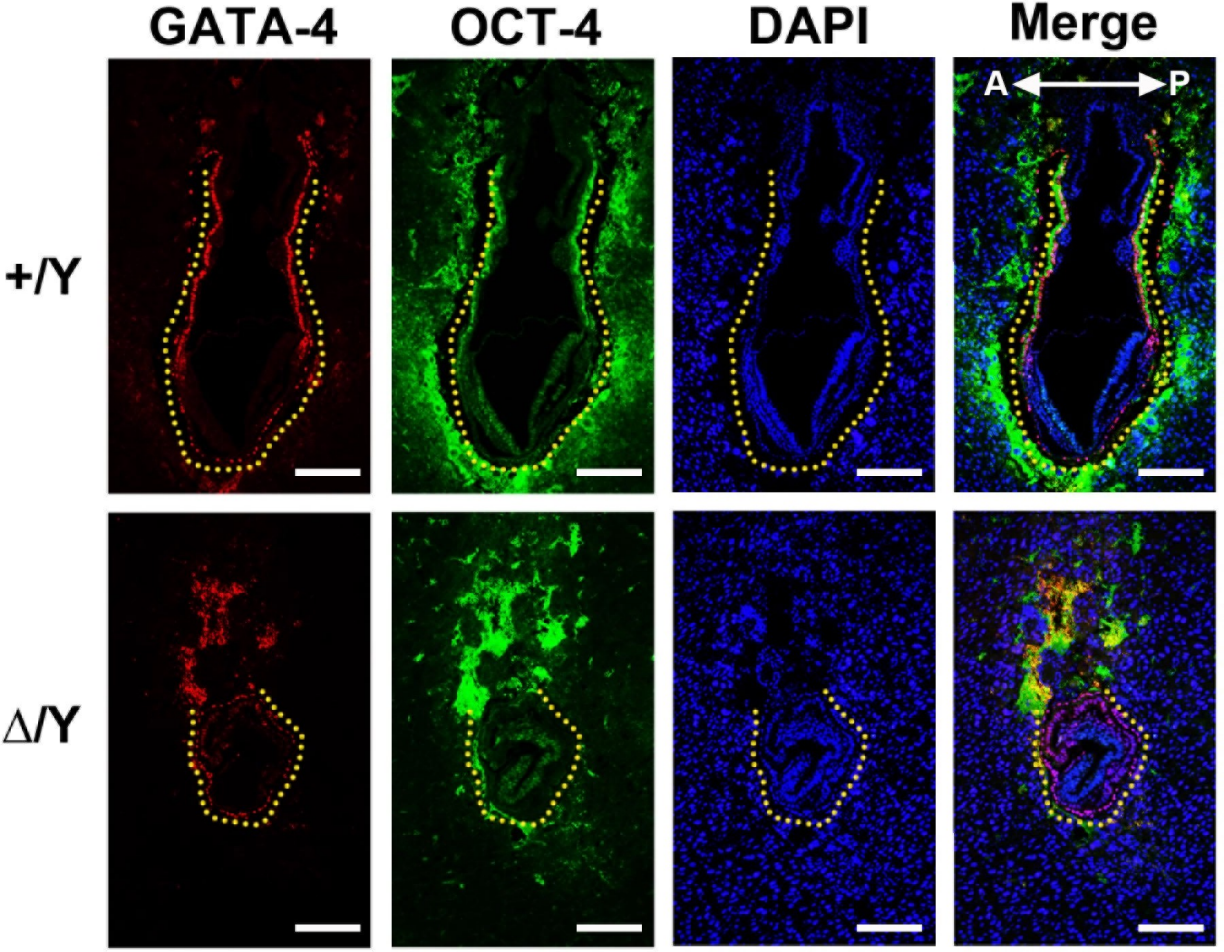
The parietal endoderm and visceral endoderm were not affected in *Cul4b*-null embryos. *Cul4b*^*+/*^*Y* and *Cul4b*^*Δ/*^*Y* embryos at E7.5 were sectioned and immunostained for GATA-4 (red), OCT-4 (green), and DAPI (blue). Dashed yellow lines indicate the cells with GATA-4 expression that are localized to regions of the parietal endoderm and visceral endoderm. All embryos were generated from *Cul4b*^*+/Δ*^ females mated with *Cul4b*^*+/*^*Y* WT males or *Cul4b*^*lox/*^*Y;Prm1-Cre* males. Scale bar: 100 μm.

In a previous study, the knock down of *Cul4b* in the extraembryonic cell line, XEN, led to an increase in apoptosis due to the accumulation of p21 protein [20]. XEN cells are derived from the distal surface of the ICM and can differentiate into the primitive endoderm and subsequently into the visceral endoderm that surrounds the entire embryo. *Gata-4* is expressed in the visceral endoderm and plays an important role in its differentiation. Therefore, we immunostained *Cul4b*^*+/*^*Y* and *Cul4b*^*Δ/*^*Y* embryos for GATA-4 to highlight the cell lineages present in the primitive endoderm [36]. GATA-4-positive cells were found in both embryonic genotypes (Fig 7). Nevertheless, fewer cells with GATA-4 immunofluorescence staining were observed in *Cul4b*^*Δ*^*/Y* embryos than in *Cul4b*^*+*^*/Y* embryos, and the overall size of the mutant egg cylinder was smaller than that in WT embryos. The result suggests that in the absence of *Cul4b*, the differentiation of the visceral endoderm remains unaffected. However, the detection of fewer visceral endoderm cells and the presence of a smaller egg cylinder in *Cul4b*^*Δ*^*/Y* embryos are consistent with results of a previous *in vitro* study of *Cul4b* knock down XEN cells [20] and the blastocyst outgrowth defect (Fig 5) observed in our study, respectively. Moreover, these data imply that the small egg cylinder is due to a proliferation defect of the *Cul4b*^*Δ*^*/Y* epiblast.

### G1/S-phase cyclins are present at lower levels in *Cul4b*-null embryos

**Fig 8:**
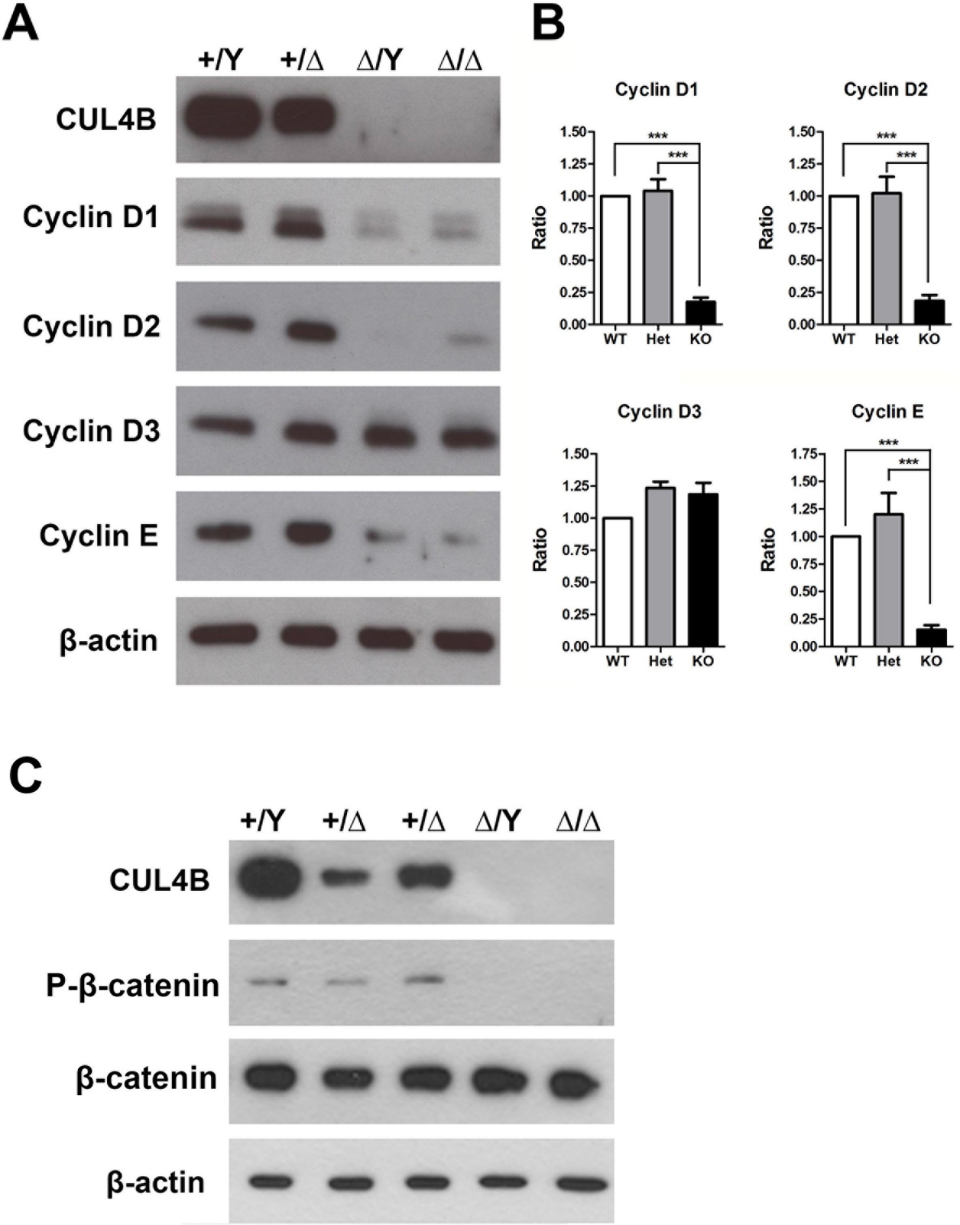
Immunoblotting of G1/S-phase cyclins in the embryo proper of *Cul4b*-null embryos at E9.5. (A) Lower expression levels of cyclins D1, D2, and E were detected in Cul4b KO embryos (*Cul4b*^*Δ/*^*Y* and *Cul4b*^*Δ/Δ*^) compared to that in *Cul4b* WT (*Cul4b*^*+/*^*Y*) and *Cul4b* Het (*Cul4b*^*+/Δ*^) embryos. The detection of β-actin was included as a loading control. Cyclin D3 showed comparable protein levels in all three embryo types. (B) Densitometry values for the protein levels of cyclins, (C) The β-catenin and phosphor-β-catenin signals detected in each of the three embryo types were normalized to the average signal for the *Cul4b* WT embryo extracts, which was set to 1, with the data obtained from three independent experiments. Each *Cul4b* KO embryo sample represents a collection of samples from three identical genotypes of E9.5 *Cul4b* KO embryos. All embryos were generated from *Cul4b*^*+/Δ*^ females mated with *Cul4b*^*+/*^*Y* WT males or *Cul4b*^*lox/*^*Y;Prm1-Cre* males. ***P < 0.001.

Based on the observation that the ICM of *Cul4b*^*Δ/*^*Y* embryos exhibits defects in proliferation and the knock down of *Cul4b* has been reported to suppress cyclin D1 protein expression [37], we hypothesized that cell cycle progression is disrupted in the absence of *Cul4b*. Therefore, we examined the expression of several G1/S-phase cyclins that positively regulate cell cycle progression in both WT and mutant embryos (Fig 8). Immunoblotting of the embryo proper showed that while cyclin D3 was present at a similar level among all of the genotypes examined, the levels of cyclins D1, D2, and E were decreased in *Cul4b*-null (*Cul4b*^*Δ/*^*Y and Cul4b*^*Δ/Δ*^) embryos compared to those in WT *Cul4b*^*+/*^*Y* embryos. Based on these results, we propose that the loss of *Cul4b* potentially interferes with cell proliferation, and this effect may directly or indirectly lead to a decrease in the expression of the cyclin proteins required for the transition of cells from the G1 to S phase of proliferation.

## Discussion

*Cul4b*-null mice undergo growth arrest and degeneration during an early embryonic stage and do not survive past E9.5 [22]. The pathogenic causes of this lethality have not been fully studied. In this study, we dissected *Cul4b-*deficient embryos at E6.5~E7.5 and observed two sets of novel findings that indicate a potential cause of death and demonstrate an important role for *Cul4b* in embryo development. The first important finding was that the loss of embryonic *Cul4b* affects the growth of the ICM *in vitro* (Fig 5) and delays epiblast development during the gastrulation period at E6.5~E7.5 *in vivo*, as highlighted by the absence of the epiblastic transcription factor, *Brachyury*, from E6.5 until E7.5 (Fig 3B). Moreover, the delay in *Brachyury* expression until E7.5 is accompanied by strong and expanded *Eomes* and *Fgf8* signals (Fig 3H and 3L). The second novel finding was that the loss of *Cul4b* resulted in an absence of the extraembryonic tissue-specific marker *Mash2* after E6.5 (Fig 6J and 6L). Previous studies have reported that *Brachyury* may function to control the ability of cells to transit out of the primitive streak [38]. Thus, epiblast cells with inappropriate *Brachyury* transcription may lose their migration. Furthermore, in previous studies, *Mash2-*null embryos exhibited severe phenotypes at E10.5 in extraembryonic tissues [39] and died at midgestation (around E10.5) due to an absence of the spongiotrophoblast layer and a reduction in the number of labyrinthine trophoblast and giant cells. Taken together, our findings suggest that *Cul4b* is important for the growth of the epiblast and the proper localization of primitive streak layer cells during E6.5~E7.5. Consequently, in the absence of *Cul4b*, delayed epiblast growth and a disorganized primitive streak could disrupt signaling between the epiblast and the chorionic ectoderm, thereby affecting ectoplacental cone development and ultimately leading to the growth retardation and lethality of *Cul4b*^*Δ/*^*Y* embryos.

Incomplete X chromosome inactivation (XCI) and reactivation of the paternal X chromosome (XCR) in extraembryonic tissues has been suggested to explain the longer survival of *Cul4b*^*Δ/+*^ embryos than that of *Cul4b*^*Δ/*^*Y* embryos. Merzouk et al. [40] showed that paternal XCI was unstable from the morula to blastocyst stage (E2.5-E3.5). A more recent work by Kobayashi et al. [41] showed that XCI could carry through to the trophectoderm of blastocysts, and be maintained after the E4.5 stage in the placenta. In terms of X chromosome reactivation (XCR), the work by Kobayashi et al., [41] seemed to indicate that XCR occurs in epiblastic cells at E4.5~E5.5 and is completed by E6.5, while XCR did not occur in the visceral endoderm and the extraembryonic lineages at E5.5. This observation seems to support our staining results (S2 Fig). CUL4B signals were barely detected in the extraembryonic tissues of *Cul4b*^*Δ/+*^ or *Cul4b*^*Δ/*^*Y* embryos by immunohistochemistry, although there is a limitation of detection.

We and others have previously shown that when *Cul4b*-null male (*Cul4b*^*Δ/*^*Y*) mice derived from *Sox2*-*Cre* (*Cul4*^*lox/*^*Y*; *Sox2*-*Cre*) mice, but not from *Protamine*-*Cre* knockout (*Cul4*^*lox/*^*Y*; *Prm1*-*Cre*) or *Zp3*-*Cre* knockout mice are mated with WT females, the offspring are viable through adulthood [20, 23, 25]. Other groups have concluded that the expression of *Cul4b* in extraembryonic tissues but not in the embryo proper plays a major role in embryo development [20, 21]. Considering that the *Sox2-Cre* line is active at E6.5 and beyond [42], and our present findings indicate a role for *Cul4b* at E6.5~E7.5, we hypothesize that the timing of *Sox2*-*Cre* expression around E6.5 may not be as precise as previously considered. Accordingly, if *Sox2-Cre* expression actually occurs at a later stage than E6.5, then its expression would be consistent with the ability of the *Cul4b* embryos to escape early embryonic lethality at E6.5~E7.5 [43]. Furthermore, we were unable to obtain *Cul4b*^*Δ/*^*Y* embryonic stem cells from *Cul4b*^*lox/*^*Y* embryonic stem cells that were transfected with a *Cre* expression plasmid to excise *Cul4b in vitro* (0/192 clones were analyzed). In combination with our finding of defective outgrowth in *Cul4b* blastocysts (Fig 5), these results suggest that *Cul4b* may play an important role in the proliferation of the ICM. A similar conclusion was made based on the observation that *Cul4b* gene-trapped mouse embryonic stem (ES) cells aggregate with tetraploid embryos, resulting in an inability of *Cul4b*-null ES cells to form chimeras [22].

The epiblast differentiates into mesoderm cells and moves through the primitive streak during the gastrulation stage. In the primitive streak of *Cul4b*^*Δ/*^*Y* embryos, an accumulation of epiblast cells was observed (Fig 4). This accumulation suggests that *Cul4b* contributes to the proper localization and further differentiation of the epiblast. Further evidence was provided from *in situ* hybridization experiments (Fig 3) that detected transcripts of *Brachyury*, *Eomes*, and *Fgf8*, which are required for the formation of the primitive streak and mesoderm in the epiblast [44]. *Brachyury* plays an important role in directing the mesoderm from the onset of gastrulation. *Brachyury* staining of E7.5 *Cul4b-*deficient embryos displayed a major phenotype of not only reduced but also expanded *Brachyury* expression (S8 Fig). Previous studies have suggested that delayed *Brachyury* expression leading to aberrant mesoderm formation resulted in a variable phenotype [45]. Thus, the variable appearance of E7.5 *Cul4b-*deficient embryos might be due to delayed *Brachyury* expression at E6.5. Nascent mesoderm cells (derived from epiblast cells) with compromised *Brachyury* function were previously shown to lose the ability to migrate away from the primitive streak [46]. The delay in *Brachyury* expression until E7.5 is accompanied by strong and expanded *Eomes* and *Fgf8* signals (Fig 3H and 3L). This abnormal expression of important mesoderm markers further confirmed that the *Eomes*-*Fgf8*-*Brachyury* signaling pathway was compromised [35]. The fibroblast growth factor (FGF) receptor is essential for the initiation of *Brachyury* expression in the primitive streak, and the loss of the FGF receptor results in abnormal gastrulation. Thus, we propose that the deletion of *Cul4b* disrupts the function of the FGF receptor in the transduction of signaling between *Fgf8* and *Brachyury*, thereby leading to overexpression feedback to the upstream inducers, *Eomes* and *Fgf8*. The resulting epiblast deficiency due to these changes could result in the early embryonic lethality of *Cul4b*^*Δ/*^*Y* embryos.

TSCs are important for the maintenance of the extraembryonic ectoderm, and these cells respond to *Fgf4* signaling from the epiblast [28, 42]. *Fgf4* signaling sustains the expression of *Cdx2* and *Eomes* in the extraembryonic ectoderm and maintains pluripotency. Consequently, embryos lacking *Fgf4* die at or before implantation [28]. In the present study, immunofluorescence staining demonstrated that CDX2 and EOMES are expressed in the extraembryonic ectoderm of *Cul4b*^*Δ/*^*Y* embryos, thereby indicating that the *Cul4b*-null epiblast can provide *Fgf4* signaling to maintain the extraembryonic ectoderm during the gastrulation stage. Supplementation with exogenous recombinant *Fgf4* and other conditional factors revealed that *Cul4b* knockdown TSCs did not show a proliferation defect *in vitro*, indicating that the intrinsic signaling pathway by which proliferation responds to growth factors is not affected (S10 Fig). In addition, there is a decrease in CDX2 and AP-2γ double-positive cells in the chorionic ectoderm of the *Cul4b*^*Δ/*^*Y* embryos due to a loss of CDX2 expression in the TSCs (S3 Fig), suggesting that the deficiency in TSCs is probably due to other signaling pathway involved in the maintenance of TSCs, such as TGF-β/actin or Nodal signaling [47, 48].

The differentiation of TSCs into giant cell progenitor cells can sustain the number of TGCs [49]. Our AP-2γ staining (Fig 2) of embryos revealed a similar amount of ectoplacental cone cells present in *Cul4b*^*Δ/+*^ and *Cul4b*^*+/*^*Y* embryos, unlike the reduced amount of ectoplacental cone cells in *Cul4b*^*Δ/*^*Y* embryos at E7.5. Considering the expression pattern of *Cul4b* observed in *Cul4b*^*Δ/*^*Y* and *Cul4b*^*Δ/+*^ embryos in the present study, the expression of *Cul4b* in epiblast cells is important for maintaining the number of TGCs. We found increased numbers of AP-2γ single-positive cells that were CDX2 negative (S3 Fig) at E6.5. We also performed a *Cul4b* knockdown experiment showing that the TSCs were minimally affected when these cells showed little CUL4B expression (S10 Fig). All these data suggested that the viability of TSCs might not be affected by the loss of *Cul4b*; however, the number of TSCs decreased, indicating that there is a deficiency in TGCs. Previous studies have reported that *Mash2-*null embryos display less severe phenotypes in the epiblast during gastrulation, yet a loss of TGC expansion leads to embryonic death between E9.5 and E10.5 [14]. Based on our *in situ* hybridization data, which showed that while we did not observe the decreased expression of *Mash2* in *Cul4b*^*Δ/+*^ embryos, *Cul4b*^*Δ/*^*Y* embryos showed a deficiency of *Mash2* transcripts at E7.5. We reason that the loss of *Cul4b* expression in the epiblast leads to deficient *Mash2* transcription in *Cul4b*^*Δ/*^*Y* embryos and a decrease in the number of secondary TGCs.

The requirement for maintenance of extraembryonic compartments is dependent on the crosstalk between extraembryonic and embryonic tissues. The deficiency of epiblasts may play an important role in the growth retardation of *Cul4b*^*Δ/*^*Y* embryos. We attempted to determine the possible mechanism by examining the potential working model of *Bmp4*, *Fgf8*, *Eomes* and *Brachyury* (Figs 3 and 6), which are required for the formation of the primitive streak and mesoderm in the epiblast [44]. In this study, we found the delayed expression of *Brachyury* at E6.5 and expanded expression of other mesoderm markers in the primitive streak at E7.5 in *Cul4b*^*Δ/*^*Y* embryos. These findings suggest a deficiency in the commencement of gastrulation and indicate a delayed switch from the self-renewal ability of the epiblast to the mesoderm specification of the primitive streak, as shown previously [50].

CUL4B is an intracellular protein, and there are numerous cellular dysfunctions due to the loss of CUL4B that could contribute to the phenotype(s) described here. We have studied many known *Cul4b* substrates, including CDT1, cyclin E, topoisomerase I, WDR5, p16, p21, p27, and histone proteins (H3K4me1, H3K4me2, H3K4me3, H3K9me2, H3K9me3, and H3K27me3) (unpublished results) and found no differences between control and mutant embryos. In this study, we tried to analyze the influence of the Wnt/β-catenin signaling pathway because CUL4B-dependent E3 ligase has been shown to positively and negatively regulate β-catenin. Moreover, the Wnt/β-catenin signaling pathway regulates *Brachyury* (see below). In *Drosophila* embryos, the loss of *Cul4b* function is associated with the upregulation of β-catenin/Armadillo [51]. On the other hand, when *Cul4b* was overexpressed or knocked down in cell culture systems, β-catenin was increased or decreased, respectively, concomitant with the up- or downregulation of cyclin D1 [52–54]. The positive regulation of *cyclin D1* expression by β-catenin was also shown in other cell type systems [55]. We showed that in *Cul4b*^*Δ/*^*Y* embryos, although total β-catenin protein was not altered, NH_2_-terminally phosphorylated β-catenin (S33/S37/T41) was decreased (Fig 8C), indicating that the Wnt/β-catenin signaling pathway might be disrupted [56, 57].

*Brachyury* is a target of the Wnt/β-catenin signaling pathway. The disruption of Wnt/β-catenin signaling by knockout of β-catenin or the β-catenin-binding protein Duplin caused early embryonic death and a lack of *Brachyury* expression [58, 59]. Additionally, studies have shown that *Brachyury* cooperates with Wnt/β-catenin signaling in differentiating mouse ES cells [38]. Therefore, we imagine that the loss of *Cul4b* might disturb the Wnt/β-catenin signaling pathway to delay the expression of *Brachyury* and induce defects in both the formation of the primitive streak and the expression of mesoderm markers. In *Cul4b*^*Δ*^*/Y* embryos, we found that the mRNA and protein levels of cyclin D1 (Fig 8 and S11 Fig) were decreased, and *Brachyury* expression was also affected (Fig 3 and S8 Fig). Altogether, we concluded that the loss of *Cul4b* might disturb the Wnt/β-catenin signaling pathway to delay *Brachyury* expression, thereby inducing defects in both the formation of the primitive streak and the expression of mesoderm markers.

In addition, *Cul4b* participates in Wnt/β-catenin signaling to control *cyclin D1* expression [55, 60–62]. During gastrulation, the transcription activities of *cyclin D1* and *cyclin D2* are initially detected in the epiblast, indicating a requirement for proliferation in the epiblast prior to differentiation. Additionally, *cyclin D1* transcripts are detected in the whole embryo proper, *cyclin D2* transcripts are specifically detected in the primitive streak, and *cyclin D3* transcripts are present only in extraembryonic tissue. After E8.5, *cyclin D3* expression is initially detected in the neurectoderm and mesoderm [63]. *Cylcin D1-3* can modulate cell cycle regulators in stem cells and influence the cell fate decisions of mesoderm cells [64]. These results imply that *Cul4b* contributes to cell cycle regulation in mesoderm formation through cyclin D proteins. Our immunoblotting data showed that *Cul4b*^*Δ/*^*Y* embryos exhibited a reduction in the protein levels of cyclin D1 and cyclin D2 but not in the levels of cyclin D3. These results are consistent with a role for the influence of *Cul4b* deficiency mainly in the gastrulation stage (E6.5~E7.5) and not in the head-fold stage (E8.5). A decrease in cyclins might indicate fewer cells entering S phase. However, the mutant embryo showed no difference in the BrdU and pHH3 assays (S4 and S6 Figs), indicating that the ratios of cells trapped in S phase and M phase, likely due to a prolonged cell cycle, are not significantly different from those of actively growing WT embryos, which is caused by a different reason (actively entering and exiting the cell cycle). Additionally, the Ki67 assay (S7 Fig) did not show a difference between different genotypes. Therefore, we conclude that in the *Cul4b*^*Δ/*^*Y* embryo, the cell proliferation events were not completely blocked, but the proliferation rate was decreased, resulting in a size reduction of the embryo proper. Moreover, all of the above findings support a *Cul4b-*β-catenin-cyclin D1-*Brachyury* signaling pathway leading to defects in proliferation and differentiation in the mesoderm during gastrulation in *Cul4b*^*Δ/*^*Y* embryos.

*CUL4B* is overexpressed in many types of human tumor cells, and this overexpression has been shown to influence the malignant behavior of these cells in regard to vascular invasion, histological differentiation, and metastasis [65–69]. When *Cul4b* was knocked down in colorectal and non-small cell lung cancer cell lines, both cell proliferation and invasion were suppressed [37, 69], concomitant with the inhibition of positive cell cycle regulators, including cyclin D1 [37, 66]. The highly proliferative phenotype of tumor cells resembles the phenotype of embryonic cells following the loss of repressive gene control. Moreover, the invasion and metastasis of tumor cells via the EMT resembles the formation of the primitive streak in an embryo [70]. *Cul4b* plays an important role in the EMT of cancer cells both *in vitro* and *in vivo* [71], and these studies also supported the important roles of *Cul4b* in primitive streak development.

Our findings suggest that *Cul4b* plays an essential role in early embryo development. Embryonic *Cul4b* is able to regulate the mesodermal marker, *Brachyury*, and the downstream β-catenin signaling pathway, both of which are required for the proliferation and differentiation of the epiblast. Furthermore, embryonic *Cul4b* appears to contribute to the process by which the chorionic ectoderm is sustained for the maintenance of TGCs, possibly via crosstalk between embryonic and extraembryonic tissues. Thus, the present findings highlight the essential role of *Cul4b* in the epiblasts of mouse embryos, warranting further studies of this protein in other organisms and under pathological different conditions.

## Supporting information

S1 Fig Breeding strategy of *Cul4b* heterozygous and null embryos. (A) Male mice carrying both the *loxP* allele and *Cre* allele (*Cul4b*^*lox/*^*Y;Prm1-Cre*) were generated by crossing *Cul4b*^*lox/+*^ female mice with transgenic mice expressing Cre under the control of the protamine 1 promoter, which mediated the excision of the floxed allele by Cre recombinase at the spermatid stage. *Cul4b*^*+/Δ*^ mice (paternal *Cul4b*-null heterozygous mice) were generated from crossing *Cul4b*^*lox/*^*Y;Prm1-Cre* male mice with WT C57BL/6 female mice. *Cul4b* KO embryos (*Cul4b*^*Δ/*^*Y* and *Cul4b*^*Δ/Δ*^) were produced from crossing *Cul4b*^*lox/*^*Y;Prm1-Cre* male mice with either WT C57BL/6 or *Cul4b*^*+/Δ*^ female mice. *Cul4b*^*Δ/+*^ mice (maternal *Cul4b*-null heterozygous mice) were generated from crossing WT C57BL/6 male mice with *Cul4b*^*+/Δ*^ mice. (B) The *Cul4b*^*lox/+*^*; Zp3-Cre* female mice were generated by mating *Zp3-Cre* female mice with the *loxP*-floxed *Cul4b* males. *Cul4b*^*Δ/+*^ mice were generated from crossing WT C57BL/6 male mice with *Cul4b*^*lox/+*^*;Zp3-Cre* female mice which carry the *Zp3-Cre* transgene for the excision of the *loxP*-floxed allele by Cre recombinase in the growing oocyte prior to the first meiotic division and production of oocytes.
(TIF)

S2 Fig Expression pattern of embryonic and extraembryonic *Cul4b* in *Cul4b^+/^Y*, *Cul4b^Δ/+^*, and C*ul4b^Δ/Y^* embryos. (A) E7.5 stage embryos of desired genotypes were stained with anti-CUL4B antibodies. The enlarged regions of the embryonic ectoderm (B) or chorionic ectoderm (*+/Y* and *Δ/+*; C) and extraembryonic ectoderm (*Δ/Y*; C) are shown. The CUL4B signal was positive in the chorionic ectoderm (ce) and in the embryonic ectoderm (ee) of *Cul4b*^*+/*^*Y* embryos and in the ee of *Cul4b*^*Δ/+*^ embryos with random inactivation of X chromosomes but not in the ce. The CUL4B signal was not detected in either the ee or extraembryonic ectoderm (exe) of *Cul4b*^*Δ/*^*Y* embryos. All embryos were generated from *Cul4b*^*+/Δ*^ females mated with *Cul4b*^*+/*^*Y* WT males or *Cul4b*^*lox/*^*Y;Prm1-Cr*e males. The scale bar is 100 μm.
(TIF)

S3 Fig Analysis of the pluripotency of trophoblast stem cells in extraembryonic tissues. Immunostaining for the pluripotent markers CDX2 (red) and extraembryonic lineage marker AP-2γ (green) was performed in WT (*Cul4b*^*+/*^*Y*) and KO (*Cul4b*^*Δ/*^*Y*) embryos at (A) E6.5 and (B) E7.5 embryo stage. Yellow dashed lines indicate the cells in the extraembryonic ectoderm or chorionic ectoderm. Nuclei are stained with DAPI (blue). All embryos were generated from *Cul4b*^*+/Δ*^ females mated with *Cul4b*^*+/*^*Y* WT males or *Cul4b*^*lox/*^*Y;Prm1-Cr*e males. Scale bar: 100 μm.
(TIF)

S4 Fig Deletion of *Cul4b* does not disrupt the proliferation of embryos. A BrdU incorporation assay was performed to determine whether proliferation deficiency is responsible for the growth retardation of KO embryos at (A) E6.5 and (B) E7.5. Both KO embryos display normal BrdU incorporation ability, similar to WT embryos. The right graph shows percentage of BrdU positive cells in *Cul4b*^*+/*^*Y*, and *Cul4b*^*Δ/*^*Y* embryos, respectively. All embryos were generated from *Cul4b*^*+/Δ*^ females mated with *Cul4b*^*+/*^*Y* WT males or *Cul4b*^*lox/*^*Y;Prm1-Cr*e males. The scale bar is 50 μm in (A) and 100 μm in (B).
(TIF)

S5 Fig Detection of apoptotic cells by TUNEL assay. (A) TUNEL-positive cells are located on the inner layers of the epiblast due to the formation of cavities in both WT and KO embryos at E6.5. (B) At E7.5, TUNEL-positive cells were increased only slightly in growth-retarded *Cul4b* KO embryos compared with those in WT embryos. The right graph shows percentage of TUNEL positive cells in *Cul4b*^*+/*^*Y*, and *Cul4b*^*Δ/*^*Y* embryos, respectively. All embryos were generated from *Cul4b*^*+/Δ*^ females mated with *Cul4b*^*+/*^*Y* WT males or *Cul4b*^*lox/*^*Y;Prm1-Cr*e males. Scale bar is 50 μm in (A) and 100 μm in (B).
(TIF)

S6 Fig Mitotic index in *Cul4b*-null embryos. Immunostaining and quantification of the mitotic index of *Cul4b* WT and KO embryos at E6.5. (A) Embryos are immunostained with OCT3/4 (red), pHH3 (green) and DAPI (blue) antibodies. (B) DAPI-normalized mitotic indexes are similar between *Cul4b* WT and KO embryos. All embryos were generated from *Cul4b*^*+/Δ*^ females mated with *Cul4b*^*+/*^*Y* WT males or *Cul4b*^*lox/*^*Y;Prm1-Cr*e males. The scale bar in (A) is 50 μm.
(TIF)

S7 Fig Proliferative ability in *Cul4b* KO embryos. Ki-67 immunohistochemical staining was performed in *Cul4b* WT and KO embryos at E7.5. Positive signals were found in the embryonic ectoderm (ee), ectoplacental cone (epc), chorionic ectoderm (ce) and extraembryonic ectoderm (exe) in both WT (*Cul4b*^*+/*^*Y*) and KO *(Cul4b*^*Δ/*^*Y*) embryos. (C) The graph shows percentage of Ki-67 positive cells in *Cul4b*^*+/*^*Y*, and *Cul4b*^*Δ/*^*Y* embryos, respectively. All embryos were generated from *Cul4b*^*+/Δ*^ females mated with *Cul4b*^*+/*^*Y* WT males or *Cul4b*^*lox/*^*Y;Prm1-Cr*e males. The scale bar is 100 μm.
(TIF)

S8 Fig Different expression patterns of *Brachyury* in *Cul4b^Δ/^Y* embryos. *Brachyury* transcripts were stained in E7.5 *Cul4b*^*+/*^*Y* and *Cul4b*^*Δ/*^*Y* embryos using whole-mount RNA *in situ* hybridization experiments. Different *Brachyury* phenotypes were detected in the nascent primitive streak of *Cul4b*^*Δ/*^*Y* embryos, including reduced expression levels (triangles) and unusual expression patterns (arrows). All embryos were generated from *Cul4b*^*+/Δ*^ females mated with *Cul4b*^*+/*^*Y* WT males or *Cul4b*^*lox/*^*Y;Prm1-Cr*e males. Scale bar: 200 μm.
(TIF)

S9 Fig Comparison of the inner cell mass between E4.5 *Cul4b* WT and KO blastocysts. Images of E4.5 *Cul4b* WT (*Cul4b*^*+/*^*Y* and *Cul4b*^*+/+*^) and KO (*Cul4b*^*Δ/*^*Y* and *Cul4b*^*Δ/Δ*^) blastocysts are shown. The inner cell mass (triangles) displayed comparable sizes between the two groups. All embryos were generated from *Cul4b*^*+/Δ*^ females mated with *Cul4b*^*+/*^*Y* WT males or *Cul4b*^*lox/*^*Y;Prm1-Cr*e males. Scale bar: 50 μm.
(TIF)

S10 Fig Immunoblotting of *Cul4b*-knockdown trophoblast stem cells. (A) Two different *Cul4b* siRNA plasmids were designed for *Cul4b* knockdown experiments. Greatly reduced expression of CUL4B protein was shown in two different *Cul4b* knockdown trophoblast stem cells compared to the naïve control (TSC) and the vector control. Detection of CUL4A and tubulin was included as a control. (B) Cell proliferative activity was estimated using the 3-(4,5-dimethylthiazol-2-Yl)-2,5-diphenyltetrazolium bromide (MTT) assay. The *Cul4b* knockdown trophoblast stem cells showed comparable proliferative activity to naïve and vector control trophoblast stem cells.
(TIF)

S11 Fig qRT-PCR of G1/S-phase cyclins in the embryo proper of *Cul4b*-null embryos at E9.5. The relative mRNA levels of cyclin D1, D2 and D3 in *Cul4b* WT (*Cul4b*^*+/*^*Y*), *Cul4b* Het (*Cul4b*^*+/Δ*^) embryos and *Cul4b* KO embryos (*Cul4b*^*Δ/*^*Y* and *Cul4b*^*Δ/Δ*^) were determined by qRT-PCR. The expression level of cyclin D1 mRNA was decreased in *Cul4b* KO embryos compared to that in *Cul4b* WT and Het embryos. The expression levels of cyclin D2 and D3 mRNA in *Cul4b* KO embryos were not significantly different from those in *Cul4b* WT and Het embryos. The ΔCT value is calculated by subtracting the GAPDH CT value from the *Cul4b* CT value. All embryos were generated from *Cul4b*^*+/Δ*^ females mated with *Cul4b*^*+/*^*Y* WT males or *Cul4b*^*lox/*^*Y;Prm1-Cr*e males. *P < 0.05.
(TIF)

S1 Table Primer sequences for genotyping and qRT-PCR. (PDF)

S1 File Supplementary materials and methods. (DOCX)
