# Supplementary material for: Embryonic Cul4b is important for epiblast growth and location of primitive streak layer cells
Source: PLoS One. 2019 Jul 1;14(7):e0219221. doi: 10.1371/journal.pone.0219221 (PMC6602292; doi:10.1371/journal.pone.0219221)
Supplement: S1 Table — (PDF) [file pone.0219221.s012.pdf]

S1 Table. Primer sequences for genotyping.

Name	Primer Sequence
in3F1	5’-CATCTTTAGC CTCTTGTGCT-3’
In3R1	5’-AAAAGCCTAC GTTTATGTGC-3’
In5R1	5’-AGCCTGGTCT ACAAAGTTGA-3’
Prm1-F	5’- GCGGTCTGGC AGTAAAAACT ATC-3’
Prm1-R	5’- GTGAAACAGC ATTGCTGTCA CTT-3’
Sry-F	5'- TGACTGGGATGCAGTAGTTC-3'
Sry-R	5'-TGTGCTAGAGAGAAACCCTG-3'
