# Supplementary material for: Embryonic Cul4b is important for epiblast growth and location of primitive streak layer cells
Source: PLoS One. 2019 Jul 1;14(7):e0219221. doi: 10.1371/journal.pone.0219221 (PMC6602292; doi:10.1371/journal.pone.0219221)
Supplement: S1 File — (DOCX) [file pone.0219221.s013.docx]

**Embryonic *Cul4b* is important for epiblast growth and location of primitive streak layer cells**

Chun-Yu Chen^1¶^, I-Shing Yu^2¶^, Chen-Hsueh Pai^1^, Chien-Yu Lin^1^, Shu-Rung Lin^3,4^, You-Tzung Chen^5^, Shu-Wha Lin^1,6 ,7*^

**Supporting information**

**Materials and Methods**

**Immunohistochemistry**

Pregnant females were killed at the desired embryonic day. Embryos within deciduas were fixed in 4% paraformaldehyde and embedded in paraffin following sectioning. Dewaxed embryo sections were boiled in 0.01 M citrate buffer, pH 6.0, and bleached with 3% H_2_O_2_. After blocking with Rodent Block M (BIOCARE MEDICAL, RBM961H), sections were incubated overnight with an appropriate amount of primary antibodies against CUL4B (HPA011880, Sigma-Aldrich) and Ki67 (ab15580, abcam) diluted in 1% FBS/PBST at 4°C. Unbound antibodies were removed by washing in PBST three times. Then detection was performed using the Super Sensitive ^TM^ Polymer-HRP IHC Detection System (BioGenex, QD430-XAK).

**Transduction of *Cul4b*-silencing lentivirus**

Two *Cul4b*-silencing lentiviruses, shCul4b-1 and shCul4b-2, and a control vector, pLAS.Void that expresses a scramble shRNA, were provided by the National RNAi Core Facility at Academia Sinica. All vectors contained a PGK-puromycin acetyltransferase expression cassette. Lentivirus transduction was performed by using 400 MOI of lentivirus with 5 μg/ml polybrene to transduce TSCs. Transduced TSCs were selected by culture medium with 2 μg/mL puromycin.

**qRT-PCR**

RNAs of embryos from desired genotype were extracted by TRIzol reagent and reverse transcribed using SuperScript III Reverse Transcriptase (Invitrogen). qRT-PCR of indicating genes was performed and expression level were normalized to GAPDH. Primer sequences according to previous studies (*Blood*. 2007 Jun 15; 109(12): 5199–5207) are shown in supplementary table S1.
